# Potential of using kaolin as a natural adsorbent for the removal of pollutants from tannery wastewater

**DOI:** 10.1016/j.heliyon.2019.e02923

**Published:** 2019-11-30

**Authors:** S. Mustapha, M.M. Ndamitso, A.S. Abdulkareem, J.O. Tijani, A.K. Mohammed, D.T. Shuaib

**Affiliations:** a Department of Chemistry, Federal University of Technology, Bosso Campus, Minna, PMB 65, Nigeria; b Department of Chemical Engineering, Federal University of Technology, Gidan Kwano Campus, Minna, PMB 65, Niger State, Nigeria; c Nanotechnology Research Group, Center for Genetic Engineering and Biotechnology, Federal University of Technology, Minna, PMB 65, Niger State, Nigeria; d Department of Chemistry and Biochemistry, North Carolina Central University, 1801 Fayetteville Street, Durham, NC, 27707, USA; e Department of Chemistry, Illinois Institute of Technology, 3101 S Dearborn Street, Chicago, IL, 60616, USA

**Keywords:** Analytical chemistry, Environmental science, Kaolin, Tannery, Adsorption, Jovanovic model, Pollutants

## Abstract

In the present work, kaolin sample from Gbako Local Government, Niger State, Nigeria was used as an adsorbent for the removal chloride, COD, BOD, sulphate, chromium, cadmium, zinc and the reduction of total alkalinity in tannery wastewater. The kaolin sample was pretreated to enhance its adsorption capacity and then characterized using X-Ray Diffraction (XRD), Fourier Transform Infrared Spectroscopy (FTIR), High Resolution Electron Microscopy (HRSEM), High Resolution Transmission Electron Microscopy (HRTEM), Energy Dispersive Spectroscopy (EDX), Selective Area Electron Diffraction (SAED) and Brunauer Emmett-Teller (BET). The specific surface area, pore volume and pore diameter of the kaolin were 17 m^2^/g, 0.018 cm^3^/g and 3.587 nm, respectively. The adsorption methods of the parameters onto the kaolin were investigated as functions of contact time, adsorbent dosage and temperature. Equilibrium isotherms for the adsorption parameters were carried out experimentally and the adsorption data correlated very well with Jovanovic and Redlich-Peterson models. Furthermore, the adsorption kinetics followed the Avrami model. From the results of the study it was established that kaolin from Gbako, Nigeria can serve as an economic, safe and effective natural adsorbent for the pollutants removal from tannery wastewater.

## Teaser

Analytical chemistry; Environmental sciencel; Kaolin; Tannery; Adsorption; Jovanovic model; Pollutants

## 1 Introduction

Environmental pollutants and their toxicity are a global concern due to their adverse effects and the severe health challenges they pose. Water pollution has become a global phenomenon, undermining the economic as well as environmental and deleterious health effect on people. Water pollution is as a result of contaminations from natural and anthropogenic activities. Contaminants originating from chemicals used in various industrial sectors (textiles, mining, electroplating, dyeing and tanning), agricultural sectors (such as pesticides, fertilizers, herbicides, fungicides) and medical sectors such as pharmaceuticals hormonal and personal care products which are extremely toxic have been released into water bodies all over the world (Awaleh and Soubaneh, 2014). Most developing countries do not have the necessary technology to remove the pollutants prior to discharge into the environment.

Specifically, tannery industries (TI) release wastewater containing phenolic compounds, sulphonated oils, chromium salts and polychlorinated biphenyls used for the conversion of raw hides into leather products (Saxena et al., 2016). According to the Nigerian Tanner Council (NTC), tannery industries in Nigeria are among the oldest industries (The News, 2014). Due to the large scale production of livestock, Nigeria is rated as one of Africa's leading leather producing countries (Nigeria Punch, April 2019). In the past, the leather industry has generated employment for young people in the North-West region of Nigeria. However, the situation has moribund due to the Federal government's banning of wet blue and crust exports. This has led to the closure of many tannery industries across the nation, making the foreign-owned tanneries to invest in new equipment and types of machinery. The number of skins produced by traditional tanners has fallen due to the use of low-grade skin and wet blue. As a result of this development, the local tannery industries have moved closer to villages and they engage in improper disposal of tannery waste into rivers and lakes. The release of these toxic substances has a negative impact on man, plant and other forms of abiotic and biotics.

Different methods have been developed and used for the treatment of wastewater. Some of the adopted techniques include centrifugation (Peeters, 2015), filtration (Cardenas et al., 2016), flotation (de Oliveira da Mota et al., 2015), oxidation and evaporation (Li et al., 2016), distillation (Ji, 2018), ion exchange (Tan et al., 2017), precipitation (Sun et al., 2017), electrolysis (Huang et al., 2016), electrodialysis (Akhter et al., 2018), adsorption (Guillaume et al., 2018), crystallization (Lu et al., 2017), micro and ultra-filtration (Pinto et al., 2017), sedimentation and gravity separation, reverse osmosis (Venzke et al., 2017) and coagulation (Mousa and Hadi, 2016). Adsorption is a process which is widely used to remove pollutants from fluids. Adsorption has proved to be a multiple sequestration method of solute separation. This technology depends on utilization of either modified or unmodified adsorbents controlled by parameters such as contact or residence time, pH, concentration, temperature and adsorbent dosage based on batch adsorption mode only (Al-Essa and Khalili, 2018).

Several kinds of non-conventional adsorbent materials have been examined for their adsorption capacity to remove pollutants but cytotoxicity and regeneration have been their major shortcomings. The most promising material used as an alternative adsorbent for these non-conventional adsorbents are clay minerals. The utilization of clay and its derivatives would solve the disposal problem, and also provide access to less-expensive materials for wastewater treatment. Due to their low production costs, clays do not need to be regenerated after use which provide more advantages in using them as adsorbent (Uddin, 2016).

The application of clay as an adsorbent for the removal of toxic pollutants from contaminated waters has been widely studied in developing countries. Kaolinite, montmorillonite, illite and bentonite are commonly used due to their high specific area, availability, stability and structural characteristics. These minerals are abundant in nature, they are non-toxic and they have significant roles in scavenging pollutants from wastewater either via ion-exchange or adsorption processes or both. Hence, they are basically used as depolluting agents. The adsorption processes, which occur on the solid surface in contact with ionic solution involves the adsorption of potential counter ions which gives the surface either positive or negative charge with respect to the charge originating from the crystal lattice. Kaolinite group is classified as 1:1 type layer silicate with a tetrahedral sheet of silica (SiO_2_) joined together with an oxygen atom and octahedral sheet of alumina (Al_2_O_3_). Kaolinite possesses high chemical stability, low expansion and cation exchange capacity. The kaolinite group is structurally divided into dioctahedral and trioctahedral minerals (Uddin, 2016).

Beneficiation is a process used to improve the quality of kaolin by removing unwanted minerals. Beneficiated kaolin was used as an adsorbent to investigate its performance in removing some pollutants from wastewater. In particular, the emphasis was placed on the reduction of BOD, COD, chloride, total alkalinity, sulphates, chromium, cadmium and zinc. To achieve this goal, the study was carried out by beneficiating the raw kaolin, followed by a comparative study of some physicochemical parameters of the raw and beneficiated kaolin, accompanied by characterization and adsorption performance. Adsorption isotherms and kinetic mechanisms of the beneficiated kaolin for the removal of the selected pollutants in tannery wastewater were investigated.

## 2 Materials and methods

### 2.1 Preparation and beneficiation of kaolin

The clay sample used in this study was obtained from a clay deposit in Gbako Local Government Area in Niger State, Nigeria located at longitude and latitude 9°24′00″N and 6°02′00″E, respectively. The collected clay samples were pre-treated to remove debris and thereafter air-dried at ambient temperature for three (3) days. The samples were crushed with ceramic pestle and mortar, passed through a 250 μm mesh sieve to obtain very fine particles and subsequently stored in a plastic container prior to analysis.

The removal of impurities from the sieved clay was done by physical separation of the dirt, followed by a wet/soaking method as described by Aroke et al. (2013). 100 g of the powdered bulk clay sample was soaked in a plastic container with 1000 cm^3^ of de-ionized water for 48 h. The resultant slurry was plunged and screened through a sieve and then allowed to settle. The mixture was soaked with deionized water and later the water was decanted and deionized water was added to the slurry for further treatment.

### 2.2 Further pre-treatment of kaolin

#### 2.2.1 Oxidation of organic matters

About 100.0 cm^3^ of 0.5 M sodium hypochlorite solution was added to the 200 cm^3^ prepared slurry (clay and de-ionized water). The mixture was stirred with a magnetic stirrer, covered and allowed to settle for 2 days. The water was decanted and the treatment was repeated twice.

#### 2.2.2 Washing and dispersing of kaolin

The resultant mixture was decanted and 10.0 cm^3^ of 0.5 M H_2_O_2_ was added for the further bleaching process. The mixture was stirred using a magnetic stirrer and allowed to settle for 2 days. The water was decanted and the bleached material was washed with de-ionized water several times, oven-dried, crushed and then stored for further analysis.

### 2.3 Batch adsorption techniques

The adsorption study was carried out using batch equilibration technique. The sorption capacity of kaolin for the studied pollutants in tannery wastewater as well as the effects of contact time, adsorbent dosage and temperature, were determined.

#### 2.3.1 Effect of the contact time

In the adsorption studies, 0.2 g of beneficiated kaolin was introduced into a 250 cm^3^ conical flask containing 40 cm^3^ of the wastewater. The mixture was shaken for 150 rpm at pre-determined time intervals (0, 5, 10, 15, 20, 25 and 30 min) for the residual concentrations of the pollutants in the solution and to attain the equilibrium time. After each time, the mixture was filtered and the exact concentration of the water indicators and heavy metals were determined using methods described by APHA (2005) and atomic absorption spectrophotometer (AAS). The experiments were conducted in duplicates.

#### 2.3.2 Effect of adsorbent dose

The effect of the dosage of kaolin (0.4, 0.6, 0.8, 1.0, 1.2 and 1.4 g) on adsorption of the parameters in 40 cm^3^ of wastewater for their optimum contact times was investigated. The corked conical flasks were shaken at 150 rpm using an orbital shaker after which the mixture was filtered and the concentration of the residual pollutants in the wastewater was determined. All the experiments were conducted in duplicates.

#### 2.3.3 Effect of temperature

The effect of temperature range from 30 to 80 °C on pollutants adsorption was investigated with 0.2 g of the adsorbent in 40 cm^3^ of wastewater in corked 250 cm^3^ conical flask. The corked conical flasks were shaken at 150 rpm using an orbital shaker to attain equilibration. The mixture was filtered and the concentrations of the remaining pollutants were determined. All the experiments were conducted in duplicates.

The removal efficiency (%) and the amounts of adsorbed at equilibrium, ${\text{q}}_{\text{e}}$ (mg/g), were calculated using Eqs. (1) and (2), respectively.

(1) $$\text{R }\left(\text{\%}\right)=\frac{{\text{C}}_{\text{o}}-{\text{C}}_{\text{e}}}{{\text{C}}_{\text{o}}}\times 100$$

(2) $${\text{q}}_{\text{e}}=\frac{{\text{C}}_{\text{o}}-{\text{C}}_{\text{e}}}{\text{M}}\text{V}$$

Where ${\text{C}}_{\text{o}}$ and ${\text{C}}_{\text{e}}$ are the initial and equilibrium concentrations (mg/dm^3^), V is the volume of the wastewater (cm^3^) and W is the weight of kaolin (g).

For batch kinetic studies, the amount of wastewater parameter adsorbed at any time, ${\text{q}}_{\text{t}}$ (mg/g), was calculated by Eq. (3).

(3) $${\text{q}}_{\text{t}}=\frac{{\text{C}}_{\text{o}}-{\text{C}}_{\text{t}}}{\text{M}}\text{V}$$

Where ${\text{C}}_{\text{t}}$ is the liquid phase concentration of wastewater at any time (mg/dm^3^).

### 2.4 Characterization techniques

The composition of kaolin was determined by X-ray diffraction (XRD) using CuK∝ radiation at 2θ angle from 100 to 90^o^. Ten milligrams (0.01 g) of the dried sample was evenly dispersed in 200 mg of spectroscopic grade KBr to record the spectra. The chemical bonds in the beneficiated kaolin were investigated by Fourier transform infrared spectroscopy (FTIR) (Thermo Scientific Nicolet iS5) in the range 4000 cm^−1^ to 500 cm^−1^. The specific area, pore volume and pore size of the beneficiated kaolin were determined by Brunauer-Emmett-Teller (BET) adsorption isotherm in nitrogen gas at -196 °C using a Novawin Quantachrome instrument. About 0.05 mg was sprinkled onto carbon adhesives tape and sputter coated with Au–Pd using a Quorum T15OT for 5 min. The microscope was operated with electron high tension at 5 kV for imaging. About 0.02 g of the synthesized samples were suspended in 10 cm^3^ of methanol and thereafter subjected to ultra-sonication until complete dispersion was achieved. Two drops of the slurry were dropped onto a holey carbon grid with the aid of a micropipette and subsequently dried by exposure to photo light prior to imaging. High-resolution scanning electron microscopy (HRSEM) and High-resolution transmission electron microscopy (HRTEM) coupled with energy dispersed spectroscopy (EDX) was performed using a Zeiss Auriga model (USA).

## 3 Results and discussion

### 3.1 Physicochemical properties of the raw and beneficiated kaolin

Table 1 shows the physicochemical properties of the raw and beneficiated kaolin. The pH values of the raw and beneficiated clay were 5.47 and 6.97 respectively. The pH value of the beneficiated clay is alkaline and this will help to promote pollutants such as heavy metal precipitation and adsorption. The cation exchange capacity (CEC) of the clay is between 8.50 and 12.15 meq/100 g (Table 1) and could be attributed to the presence of inorganic/organic matter; thus, making the beneficiated kaolin play an important role in the adsorption of heavy metal ions. The Electrical Conductivity (EC) of the beneficiated clay is higher than that of the raw sample. The higher EC of the beneficiated kaolin than that of the raw sample implies that the beneficiated clay has more dissolved salts which could allow for the removal of some toxic metal ions. Also, its high porosity could provide an excess interlayer spacing making it an effective adsorbent for the removal of pollutants.

**Table 1:** The physicochemical properties of the raw and beneficiated kaolin.

Parameter	Raw	Beneficiated
Colour/Texture	White/Fine	White/Finer
pH	5.47 ± 0.24	6.97 ± 0.12
Cation Exchange Capacity (CEC) (meq/100 g)	8.50 ± 0.52	12.15 ± 0.91
Electrical Conductivity (EC) (μS/cm)	198 ± 0.31	298 ± 0.82
Aluminum (Al) (mg/kg)	2606 ± 0.17	2700 ± 0.34
Iron (Fe) (mg/kg)	3972 ± 0.15	9788 ± 0.40
Cadmium (Cd) (mg/kg)	ND	ND
Lead (Pb) (mg/kg)	0.002 ± 0.01	ND

**Key:** ND = Not detected.

### 3.2 Characterization of kaolin

#### 3.2.1 XRD analysis

**Figure 1:**
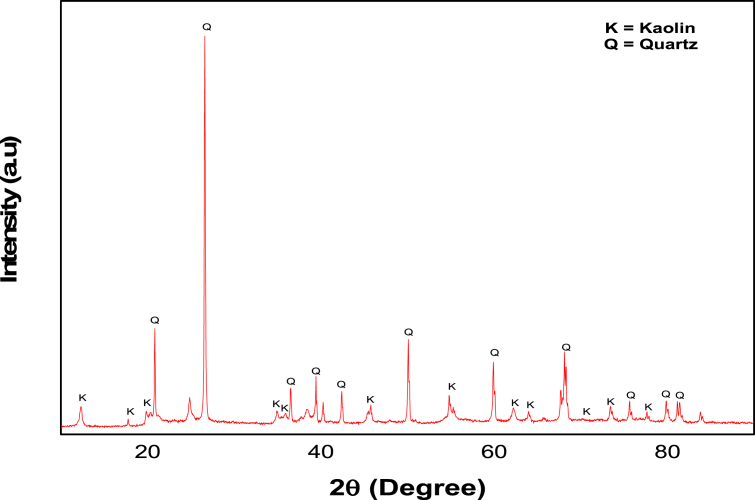
XRD pattern of the beneficiated kaolin.

The mineralogical composition of the beneficiated clay was determined by XRD. The diffractogram at 2θ was between 20 to 90^o^. The XRD pattern of the beneficiated clay is as shown in Figure 1. It was noted that the peak of quartz is highly intense and well-promoted aggregate of kaolinite are found. The JCPDS file revealed the presence of main diffraction peaks at 2θ: 12.40^o^, 19.85^o^, 24.95^o^, 36.07^o^, 46.59^o^, 54.61^o^ and 73.77^o^ which correspond to the crystallographic orientations of (001), (020), (002), (200), (221), (150) and (-402) respectively. Similar diffraction peaks were also reported by Zen et al. (2018). The kaolin sample showed predominant phases as kaolin and quartz, which are commonly found in kaolin as one of the major compositions.

#### 3.2.2 HRSEM analysis

**Plate 1:**
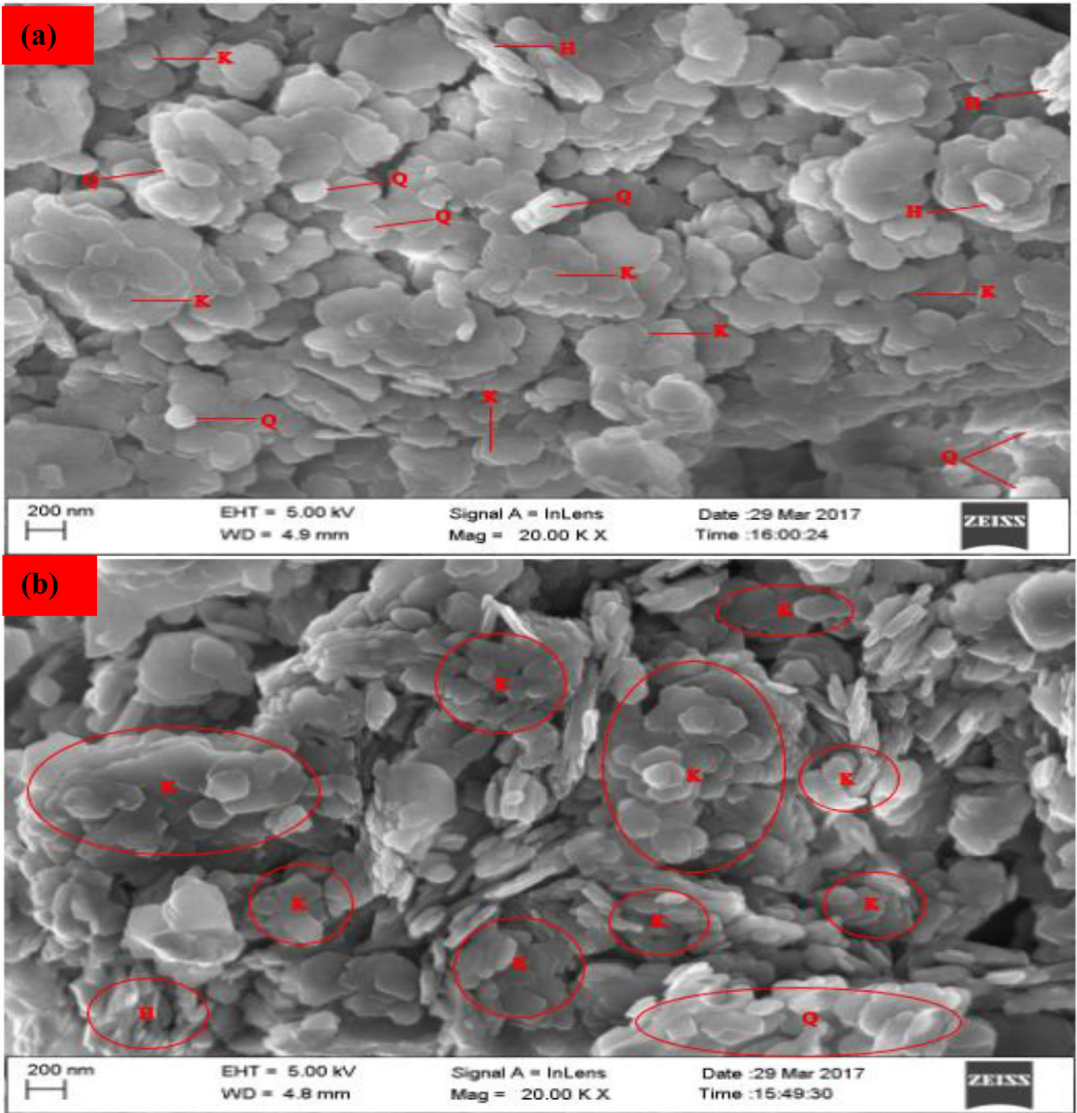
SEM analysis: (a) Raw and (b) Beneficiated kaolin.

The results of the surface morphologies of the raw and beneficiated kaolin analyzed by High-Resolution Scanning Electron Microscopy depicted in Plate 1 shows that the raw kaolin contains a mixture of halloysite, quartz and kaolin forming dense aggregate texture. The long tubes were identified as halloysite. As shown in Plate 1 (a), the tubes of halloysite are not randomly oriented but roughly packed on the surface of the clay. It was observed that small flakes with hexagonal structures were arranged in face-to-face patterns compared to the well crystalline pseudohexagonal edges of kaolinite as well as plate-like edged kaolinite particles seen in Plate 1 (a). In Plate 1 (b), kaolin particles are quite prominent with more distribution, fragmentation and fewer aggregations. This could be due to the treatment methods like washing and calcination (450 °C), leading to the removal of impurities such as organic carbon and matter. At this stage, the most stable phase of kaolinite instead of silicate was formed. This results in the formation of a more porous structure of the kaolinite lattice. Therefore, the occurrence of negative charges on the basal surface of this structure will be responsible for the adsorption of cations.

#### 3.2.3 FTIR analysis

**Figure 2:**
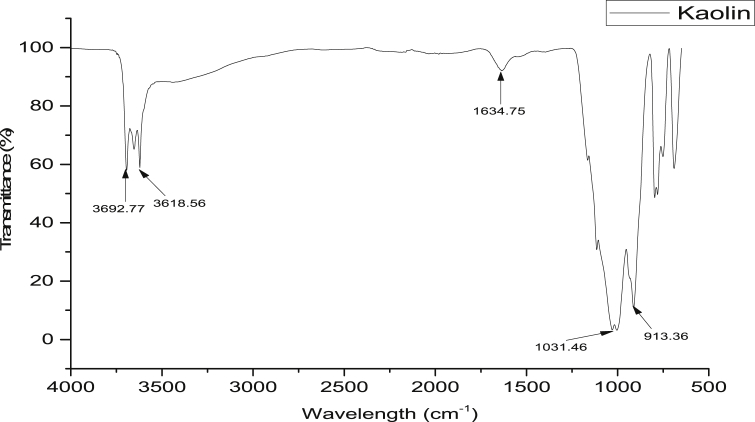
FTIR analysis of the beneficiated kaolin.

The FTIR spectrum of the beneficiated kaolin as shown in Figure 2 reveals the absorption bands 3692 and 3618 cm^−1^ assigned to be OH stretching vibrations while absorption bands at 1634 cm^−1^, 1031 cm^−1^, 1050 cm^−1^ and 913 cm^−1^ are H–O–H interlayer, Si–O–Si group of the tetrahedral sheet, Si–O stretching vibration and Al–OH, respectively. These functional groups are common to kaolinite minerals. The peaks at 750 and 467 cm^−1^ correspond to quartz while the bands at 430 cm^−1^ in the kaolin correspond to the stretching vibration modes of Si–O, Al–O and Si–O–Si bonds. This information provides the surface functional groups that will take part in the adsorption processes on the surface of kaolin.

#### 3.2.4 HRTEM analysis

**Plate 2:**
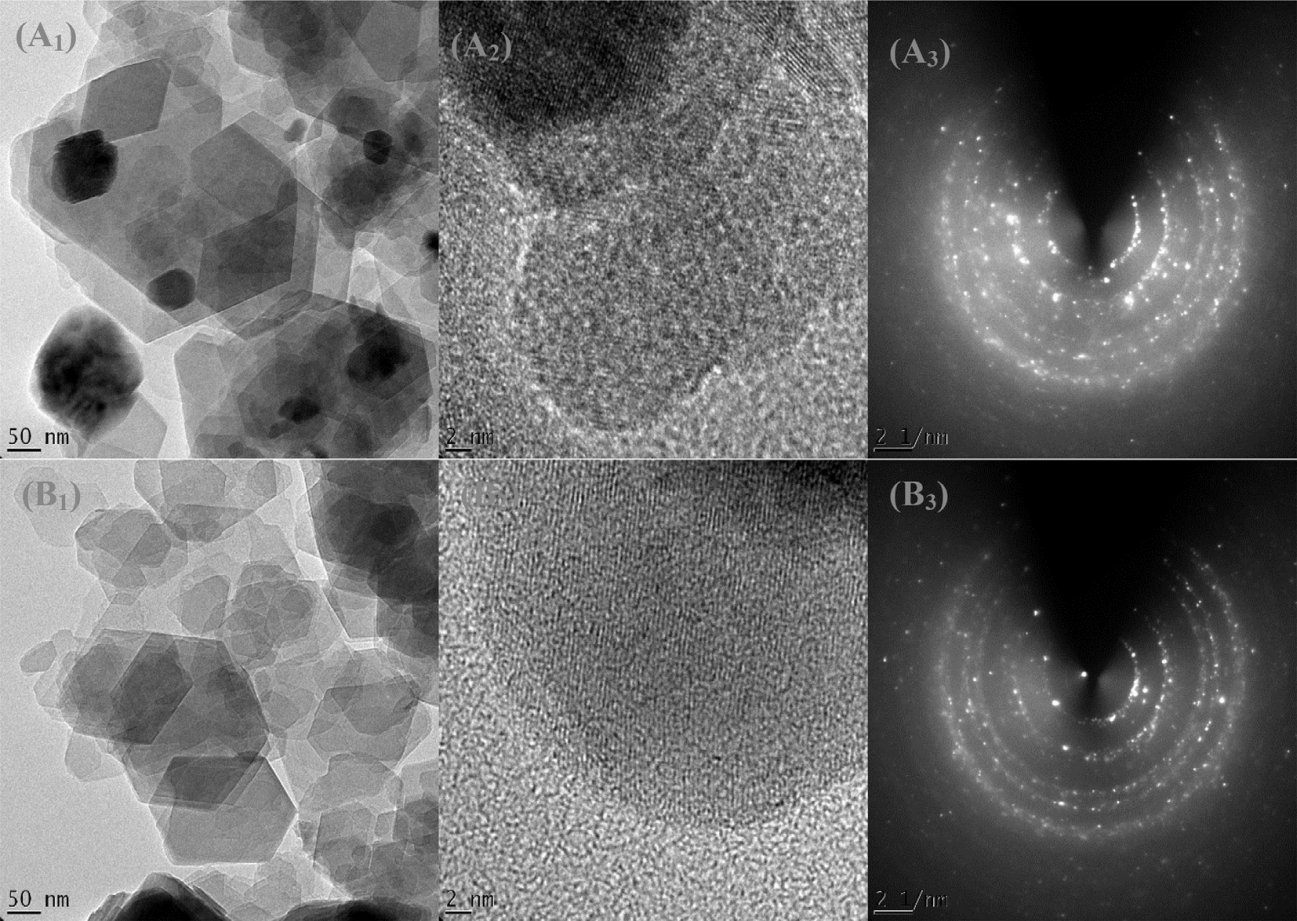
HRTEM and SAED patterns of the raw (A_1-3_) and beneficiated kaolin (B_1-3_).

The microstructures of the raw and beneficiated kaolin revealed a pseudo-hexagonal structure of kaolinite as presented in Plate 2 (A1-3) and (B1-3) from HRTEM analysis, respectively. The purification of kaolin resulted in the improvement in the crystallinity of the kaolinite from the EDX result (Figure 4) obtained. The presence of cross-fringes indicates the edge observation that the inter-network layers are coherently stacked and the stacking is ordered mostly within those domains.

**Figure 3:**
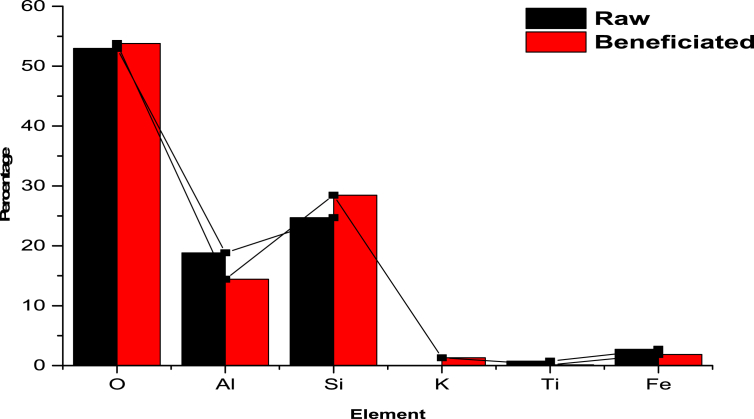
EDX analysis of raw and beneficiated kaolin.

Based on the Selected Area Electron Diffraction (SAED) investigation on the kaolin samples in this study, turbostratic patterns were observed over the aggregation of amorphous structure/particles that were randomly oriented to each other. This indicates that the particles are kaolinite phases with high crystallinity as shown in Plate 2 with dotted concentric rings assigned to quartz and halloysite forms. The EDX spectra reveal the existence of the following elements: O, Al, Si, K, Ti and Fe (Figure 3). Although, both samples exhibit similar morphologies, however, they differ based on the EDX results presented in Figure 3. It shows that the major difference may be attributed to the percentage reduction of Fe and the additional presence of K in the raw kaolin. These results suggest that there is a possibility of cations exchange between exchangeable cation in the kaolin samples and metal ions in solution, thus enhancing high adsorption capacity and removal efficiency of the metal ion by the kaolinite samples. These results are consistent with the XRD and FTIR results.

#### 3.2.5 BET analysis

**Figure 4:**
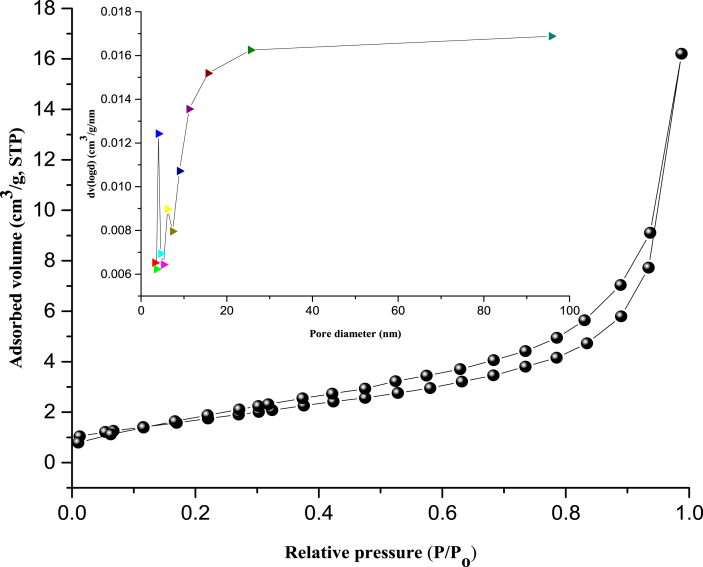
BET Analysis for beneficiated kaolin.

The quasi-overlapping adsorption-desorption curves and the pore size distribution (inlet) of the beneficiated kaolin are presented in Figure 4. The N_2_ adsorption-desorption isotherm curve of the sample can be classified as Type IV, which belongs to a Type H3 hysteresis loop indicating a purely mesoporous material with small pore size. The BET surface area and pore volume of kaolin according to Barrette-Joyner-Halenda method were 17 m^2^/g and 0.018 cm^3^/g, respectively. The beneficiated kaolin has a sharp pore distribution peak of 3.587 nm, considered to be mesoporous.

### 3.3 Physicochemical properties of tannery wastewater

The physical and chemical properties of the analyzed tannery wastewater with respect to Biological Oxygen Demand (BOD), Chemical Oxygen Demand (COD), colour, odour, pH, electrical conductivity, chloride, nitrate, nitrite, sulphate, TSS, TDS, total hardness, total alkalinity, total organic carbon and carbon dioxide are presented in Table 2.

**Table 2:** Physicochemical properties of tannery wastewater.

Parameter	Recorded value	WHO (2002)	NESREA (2009)
Colour	Dark brown		
Odour	Objectionable		
pH	5.84 ± 0.02	5–5.9	
Chemical oxygen demand (COD) (mg/dm^3^)	1988.60 ± 0.23	250	60
Biological oxygen demand (BOD) (mg/dm^3^)	625.30 ± 0.10	30	30
Electrical conductivity (μS/cm)	1647.00 ± 0.20	1200	
Chloride (mg/dm^3^)	7580.50 ± 0.42	1000	250
Nitrate (mg/dm^3^)	118.30 ± 0.16		
Sulphate (mg/dm^3^)	2944.00 ± 0.22		
Total suspended solids (TSS) (mg/dm^3^)	438.00 ± 0.30	60	
Total dissolved solids (TDS) (mg/dm^3^)	724.00 ± 0.10		
Nitrite (mg/dm^3^)	16.5 ± 0.19	3	0.5
Total hardness (TH) (mg/dm^3^)	1500.00 ± 0.61		
Total alkalinity (TA) (mg/dm^3^)	2200.00 ± 0.91		
Total organic carbon (TOC) (%)	1.41 ± 0.25		
Carbon dioxide (mg/dm^3^)	48.27 ± 0.15		
Zinc (mg/dm^3^)	2.15 ± 0.10	1.0	
Lead (mg/dm^3^)	1.70 ± 0.13	0.1	
Cadmium (mg/dm^3^)	3.23 ± 0.50	2.0	
Copper (mg/dm^3^)	0.71 ± 0.20	0.1	
Nickel (mg/dm^3^)	0.67 ± 0.33	3	
Chromium (mg/dm^3^)	8.30 ± 0.28	2	1.5–2.0
Manganese (mg/dm^3^)	0.22 ± 0.10		
Aluminium (mg/dm^3^)	2.98 ± 0.14		
Iron (mg/dm^3^)	7.20 ± 0.60	10	

The pH influences other physicochemical parameters especially the metal ions present in the wastewater. The pH of the tannery wastewater is 5.94 which is slightly acidic and may be as a result of sulphate and chloride used during the tanning process. Although, the pH value is within the range of the permissible value for industrial wastewater set by World Health Organization (WHO) (2002) which is 5–5.9. The discharge of this untreated wastewater into water bodies may be detrimental to aquatic life such as zooplankton and fish.

According to Table 2, it can be seen that the amount of COD in the tannery wastewater is 1988.60 mg/dm^3^ which is above the permissible levels given by World Health Organization (WHO) (2002) and National Environmental Standards and Regulations Enforcement Agency (NESREA) (2009). This indicates that a high amount of chemicals was used during different tanning processes. COD is the amount of oxygen used by the oxidizable matter or organic impurities in the wastewater and the high COD content from the wastewater could affect the gill breathing mechanism of aquatic organisms as a result of the reduction in available dissolved oxygen.

The BOD level obtained for the wastewater is 625.30 mg/dm^3^ which is above the permissible limit of 60 mg/dm^3^ set by NESREA (2009) for effluent discharged as depicted in Table 2. The high BOD content could be due to the presence of organic substances responsible for the consumption of oxygen in the wastewater. Furthermore, the presence of organic matter could promote anaerobic action when discharged into the water bodies. The high level of BOD could be related to the unabsorbed chemicals in the tanning process.

The EC value of the wastewater is 2647 μS/cm as shown in Table 2. The EC analysis measures the number of dissolved ions in wastewater. The obtained EC value is greater than the 1200 μS/cm standard value by WHO (2002). This indicates that the high level of EC might be attributed to various soluble salts used during the tanning process.

The concentration of chloride in the tannery wastewater is 7580.50 mg/dm^3^ as shown in Table 2. The value exceeds the permissible limit of 1000 and 250 mg/dm^3^ of WHO (2002) and NESREA (2009), respectively. The chloride level gives insight into the salinity of the wastewater sample. The high level of chloride in the wastewater could be due to the sodium chloride used during soaking and pickling stages in the tanning process. On discharge of this wastewater into water bodies, they may become polluted, limiting the quality of water for drinking and irrigation and may cause harm to zooplankton.

The TSS value in the wastewater sample is 438 mg/dm^3^ and is higher than the permissible TSS level of 60 mg/dm^3^ set by WHO (2002). According to different scholars, TSS in wastewater (100 mg/dm^3^ is weak, > 100 mg/dm^3^ but <220 mg/dm^3^ is medium and >220 mg/dm^3^ is strong. The result of this study shows that the tannery wastewater could be categorized as strong wastewater. The level of TSS in tannery wastewater solemnly depends on the quality of hides and skins. The level of TSS might be attributed to the presence or accumulation of residues from the discharged chemicals and fine leather particles during processing. The presence of TSS in water bodies could lead to high turbidity which might cause low photosynthesis and respiration in aquatic systems. Thus, the discharge of this wastewater into water bodies surrounding the tannery could lead to these environmental problems mentioned above.

In this study, the level of TDS in the tannery wastewater is 724 mg/dm^3^ as presented in Table 2 and the level of TDS may affect the aesthetic content of water via increased in turbidity. The high content of TDS could be due to the presence of chemical compounds such as carbonates, chlorides and sulphates in the wastewater. This indicates that the direct discharge of this tannery wastewater could limit the availability of good water for agricultural and domestic uses.

### 3.4 Adsorption studies

#### 3.4.1 Effect of contact time

**Figure 5:**
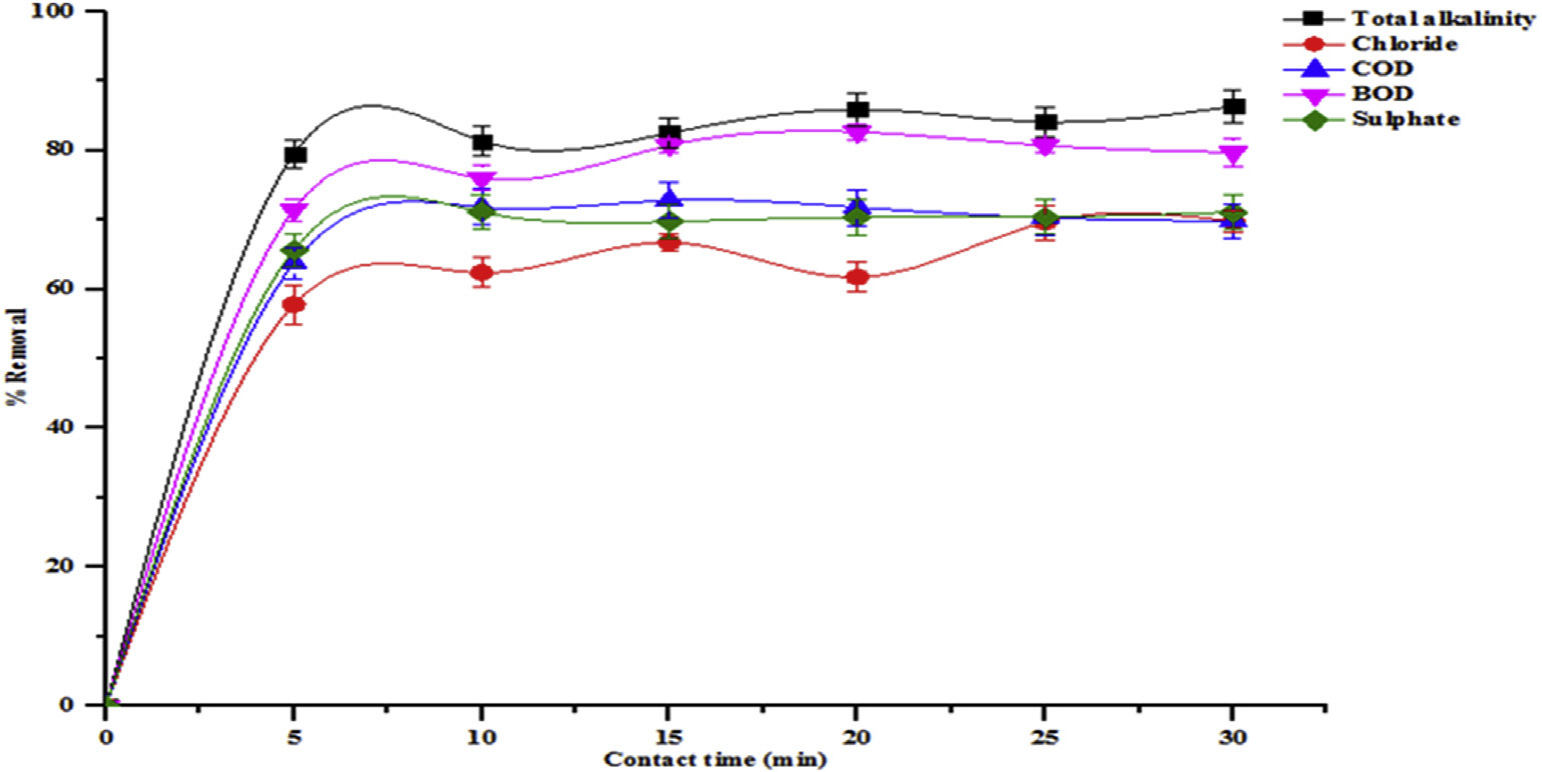
Effect of contact time on percentage removal of total alkalinity, chloride, COD, BOD and sulphate onto the beneficiated kaolin (conditions: adsorbent dose 0.2 g, agitation speed 150 rpm, temperature 29 °C and pH 5.84).

**Figure 6:**
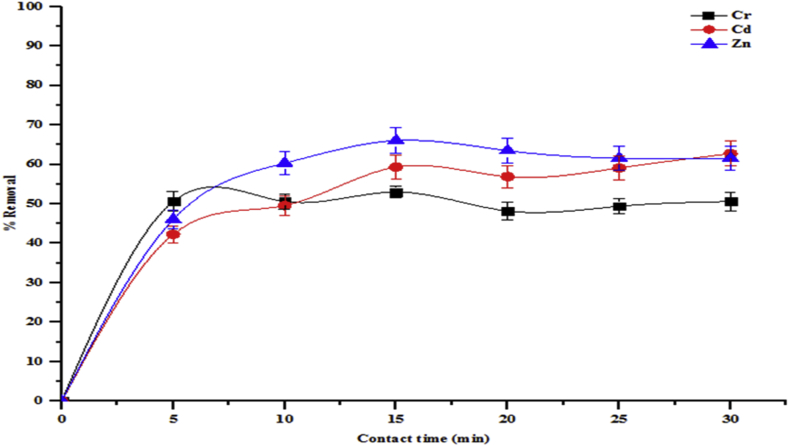
Effect of contact time on percentage removal of Cr, Cd and Zn onto the beneficiated kaolin (conditions: adsorbent dose 0.2 g, agitation speed 150 rpm, temperature 29 °C and pH 5.84).

The contact time required for the effective removal of pollutants by adsorbents is important in order to determine when equilibrium time is achieved. The effect of contact time on the reduction of total alkalinity, chloride, COD, BOD, sulphate, Cr, Cd and Zn in the tannery wastewater unto the beneficiated kaolin were determined as shown in Figures 5 and 6. The rate of removal of all parameters from solution was initially rapid and then diminished gradually until a maximum time beyond which there were no significant increases in the removal rates. Equilibrium times were achieved for the adsorption of all the pollutants using the beneficiated kaolin. The equilibrium percentage adsorptions were sulphate (71.11 %), COD (72.83 %), chloride (66.75 %), total alkalinity (85.91 %) and BOD (82.65 %) at contact times of 10, 15 and 20 min, respectively. For the heavy metals removal, the adsorption of Cr, Cd and Zn attained equilibria of 53.01 %, 59.34 % and 66.03 % respectively based on 15 min contact times. From this result, it can be deduced that the optimum time for the heavy metals removal was uniform at 15 min contact time. At this point, the rates of adsorption equal the rates of desorption. Further increase in contact time showed a slight decrease in the percentage of metal removal. The initial fast adsorption rates are due to the availability of abundant active sites on the surface of the adsorbents. The adsorption rapidly occurs and this is controlled by the diffusion process from the bulk of the solution to the adsorbent surfaces. The slow uptake at the later stages is probably due to an attachment-controlled process caused by less available active sites for adsorption, while the slight decrease in percentage removal with further increase in contract time may be due to the saturation of the surface of the adsorbent with metal ions followed by subsequent adsorption and desorption processes.

#### 3.4.2 Effect of dosage

**Figure 7:**
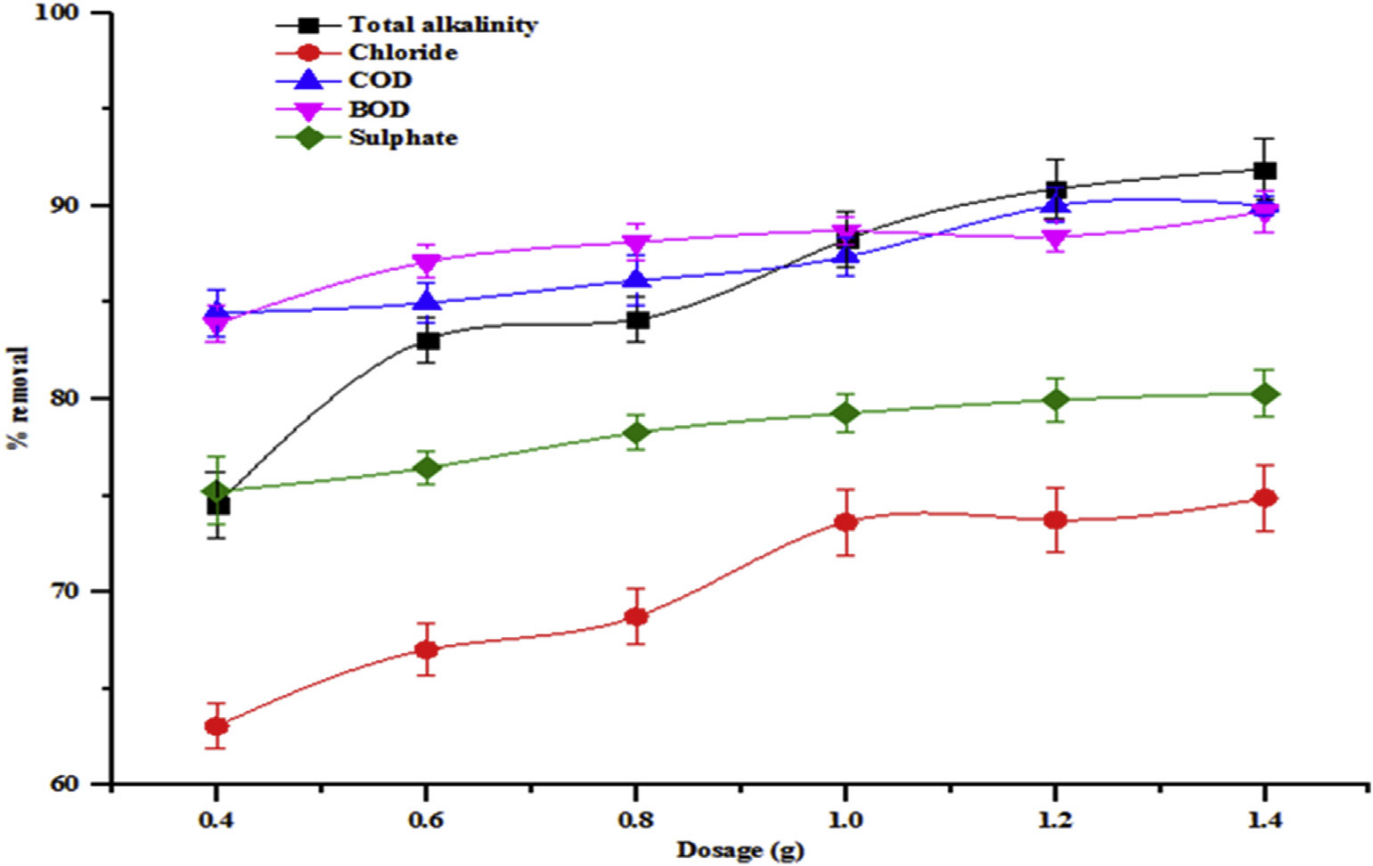
Effect of adsorbent dosage on percentage removal of total alkalinity, chloride, COD, BOD and sulphate onto the beneficiated kaolin (conditions: agitation speed 150 rpm, temperature 29 °C and pH 5.84).

**Figure 8:**
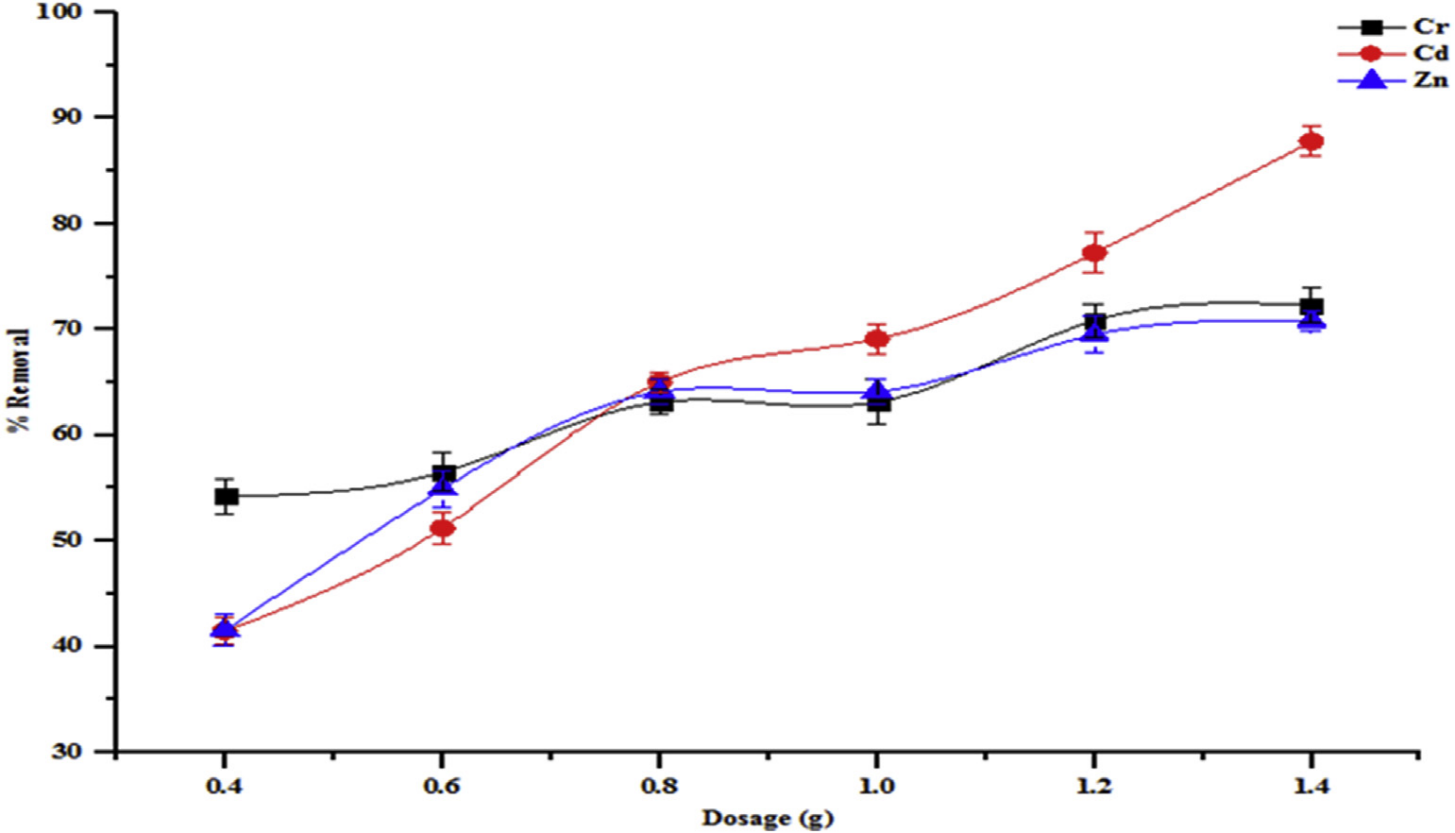
Effect of adsorbent dosage on percentage removal of Cr, Cd and Zn onto the beneficiated kaolin (conditions: agitation speed 150 rpm, temperature 29 °C and pH 5.84).

Adsorbent dosage is a very important parameter for the adsorption of pollutants on adsorbents. The effect of adsorbent dose on the percentage removal of some parameters like total alkalinity, chloride, BOD, COD and sulphate from wastewater are presented in Figure 7. While the removal of Cr, Cd and Zn from the wastewater are shown in Figure 8. The effects of dosage on the adsorption of all the parameters adsorbed onto beneficiated kaolin were investigated from 0.4 to 1.4 g. It is noteworthy that there were increases in the percentage removal of all the parameters with the increase in adsorbent dosage amount used. Increase in adsorption of total alkalinity from 74.50 to 91.91 %, chloride from 63.06 to 74.87 %, COD from 84.47 to 90.40 5, BOD from 83.93 to 89.73 and sulphate from 75.24 to 80.30 % were obtained. A similar trend was observed for the adsorption of the heavy metals unto kaolin: Cr (54.22–72.29 %), Cd (41.46–87.81 %) and Zn (41.59–70.79 %). At a lower dosage amount, the rate of adsorption is influenced by inter ionic competition among the metal ions which is due to the presence of small surface area. At a higher dosage amount, the adsorption process increased. This is as a result of an increase in the availability of active binding sites and large surface area of the adsorbent. Thus, this affected the removal efficiency of the pollutants from the solution by the adsorbent. This could also be inferred to be due to the availability of vast exchangeable sites for adsorption. The adsorption trend of the water characteristics studied on the adsorbent was in the order total alkalinity>COD>BOD>sulphate>chloride while that for heavy metals was Cd>Cr>Zn. It is obvious that the affinity of the investigated kaolin towards the studied pollutants in wastewater depends on their concentrations and the number of available binding sites on kaolin.

#### 3.4.3 Effect of temperature

**Figure 9:**
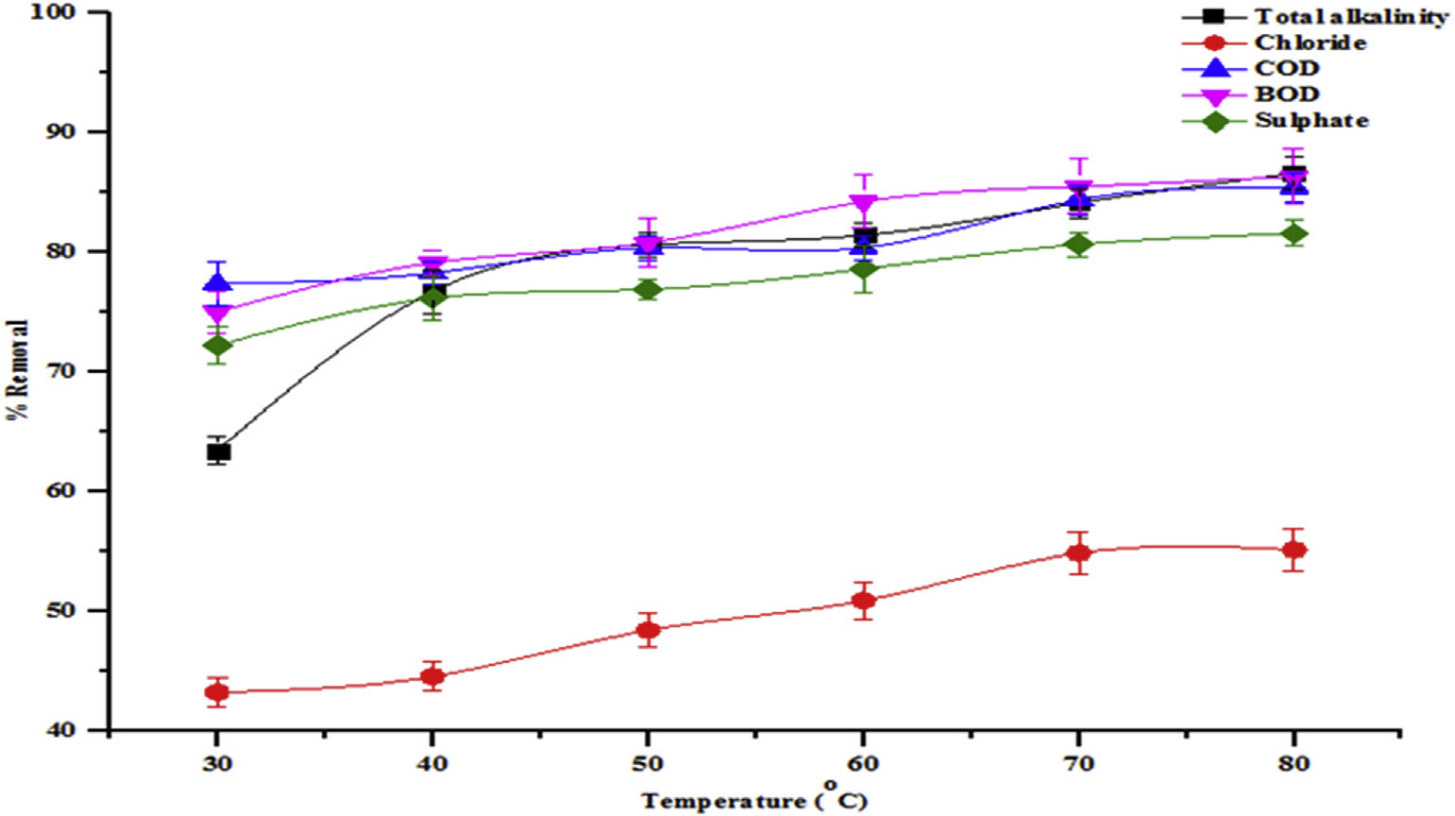
Effect of temperature on percentage removal of total alkalinity, chloride, COD, BOD and sulphate onto the beneficiated kaolin (conditions: agitation speed 150 rpm, dosage 0.2 g and pH 5.84).

**Figure 10:**
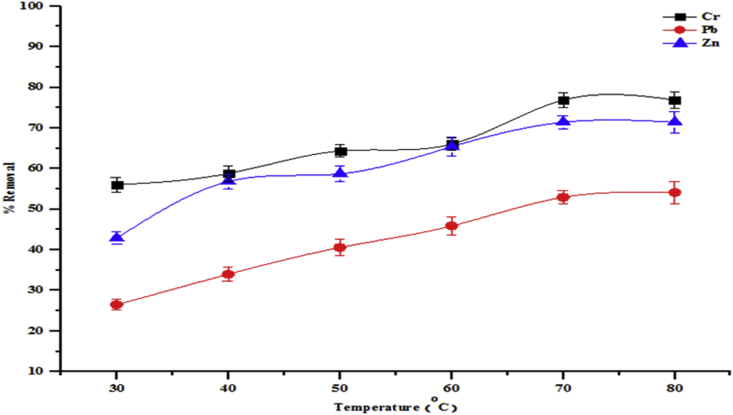
Effect of temperature on percentage removal of Cr, Cd and Zn onto the beneficiated kaolin (conditions: agitation speed 150 rpm, dosage 0.2g and pH 5.84).

The effects of temperature on the adsorption isotherms were investigated in the temperature range of 30–80 °C. Temperature is a highly important parameter in the adsorption process. The influence of temperature on the removal of total alkalinity, chloride, COD, BOD and sulphate by beneficiated kaolin is shown in Figure 9. While Figure 10 shows the adsorption of heavy metals onto kaolin. As can be seen from the figures, the percentage adsorption of all parameters in the wastewater onto the adsorbent increased with increase in temperature. With increase in temperature, the sequestration of total alkalinity, chloride, COD, BO, sulphate, Cd, Cr and Zn onto the adsorbent increased from 63.42 to 86.56 %, 43.26–55.14 %, 77.34–85.38 %, 75.02–85.38 %, 75.02–86.31 %, 58.54–89.43 %, 56.02–76.87 % and 42.85–71.43 %, respectively. The increase in adsorption may be due to the increase in kinetic energy at higher temperatures. This leads to an increase in the adsorption rates rather than desorption. Furthermore, at higher temperatures, the interaction between the pollutants and the active sites of the adsorbents becomes stronger due to the increase in the dissolution or solubility of the pollutants in the solution with temperature.

### 3.5 Adsorption isotherm

It is important to understand the equilibrium isotherm application for effective analysis and the design of adsorption process. Adsorption isotherms provide data for the evaluation of adsorption process as a unit operation. In this present study, the Halsey, Jovanovic, Redlich-Peterson and Flory-Huggins isotherms were employed to explain the experimental data. The correlation coefficient (R^2^) was used to determine the well-fitted model and the closest R^2^ to 1. Tables 3 and 4 present the calculated equilibrium isotherm parameters.

**Table 3:** The parameters of Jovanovic, Halsey, Flory-Huggins and Redlich-Peterson isotherms of BOD, total alkalinity, COD, chloride and sulphate removal using kaolin at dosage (0.2 g) and pH (5.84).

Isotherm		Parameter				
		Total Alkalinity	Chloride	COD	BOD	Sulphate
Halsey	${\text{n}}_{\text{H}}$	3.175	0.917	4.474	4.360	3.399
	${\text{I}}_{\text{n}}{\text{K}}_{\text{H}}$	24.646	14.712	31.756	24.885	27.296
	${\text{R}}^{2}$	0.95226	0.99501	0.99434	0.98658	0.99079
Jovanovic	${\text{q}}_{\text{max}}$	459.896	1694.21	369.814	125.015	596.453
	${\text{K}}_{\text{j}}$	6.156	2.046	5.568	1.690	3.973
	${\text{R}}^{2}$	0.99775	0.99871	0.99979	0.99949	0.99951
Redlich-P	β	1.315	2.023	1.224	1.229	1.294
	A	2.349×10^2^	3.449×10^6^	1.210×10^3^	3.011×10^2^	3.074×10^3^
	${\text{R}}^{2}$	0.99716	0.99871	0.99981	0.99953	0.99952
Flory-H	n	-0.315	-1.023	-0.224	-0.229	-0.294
	${\text{K}}_{\text{FH}}$	2.150×10^−5^	3.218×10^−5^	3.315×10^−4^	8.793×10^−4^	1.691×10^−4^
	${\text{R}}^{2}$	0.95221	0.99501	0.99627	0.98658	0.99079

**Table 4:** The parameters of Jovanovic, Halsey, Flory-Huggins and Redlich-Peterson isotherms of Cr, Cd and Zn removal using kaolin at dosage (0.2 g) and pH (5.84).

Isotherm		Parameter		
		Chromium	Cadmium	Zinc
Halsey	${\text{n}}_{\text{H}}$	2.129	3.155	1.424
	${\text{I}}_{\text{n}}{\text{K}}_{\text{H}}$	4.258	6.694	2.070
	${\text{R}}^{2}$	0.98028	0.93381	0.95383
Jovanovic	${\text{q}}_{\text{max}}$	106.965	32.246	60.390
	${\text{K}}_{\text{j}}$	0.146	0.938	0.390
	${\text{R}}^{2}$	0.99760	0.99671	0.99169
Redlich-P	β	1.470	1.317	1.702
	A	17.460	0.120	4.282
	${\text{R}}^{2}$	0.99796	0.99596	0.99189
Flory-H	n	-0.470	-0.317	-0.702
	${\text{K}}_{\text{FH}}$	4.689×10^−2^	3.709×10^−1^	9.380×10^−3^
	${\text{R}}^{2}$	0.98028	0.93381	0.95383

#### 3.5.1 Halsey isotherm

Halsey model (1948) was used to evaluate the multilayer adsorption at relatively large distances from the surfaces of the adsorbents. The equation of the adsorption isotherm presented as:

(4) $${\text{q}}_{\text{e}}=\frac{1}{{\text{n}}_{\text{H}}}{\text{I}}_{\text{n}}{\text{K}}_{\text{H}}-\frac{1}{{\text{n}}_{\text{H}}}{\text{lnC}}_{\text{e}}$$

Where ${\text{n}}_{\text{H}}$ and ${\text{I}}_{\text{n}}{\text{K}}_{\text{H}}$ are Halsey isotherm constants were used and these were determined from the slope and intercept of the linear plot of ${\text{q}}_{\text{e}}$ as the ordinate and ${\text{lnC}}_{\text{e}}$ as the abscissa in the temperature range of 30–80 °C, constant pH and adsorbent dosage of 0.2 g.

**Figure 11:**
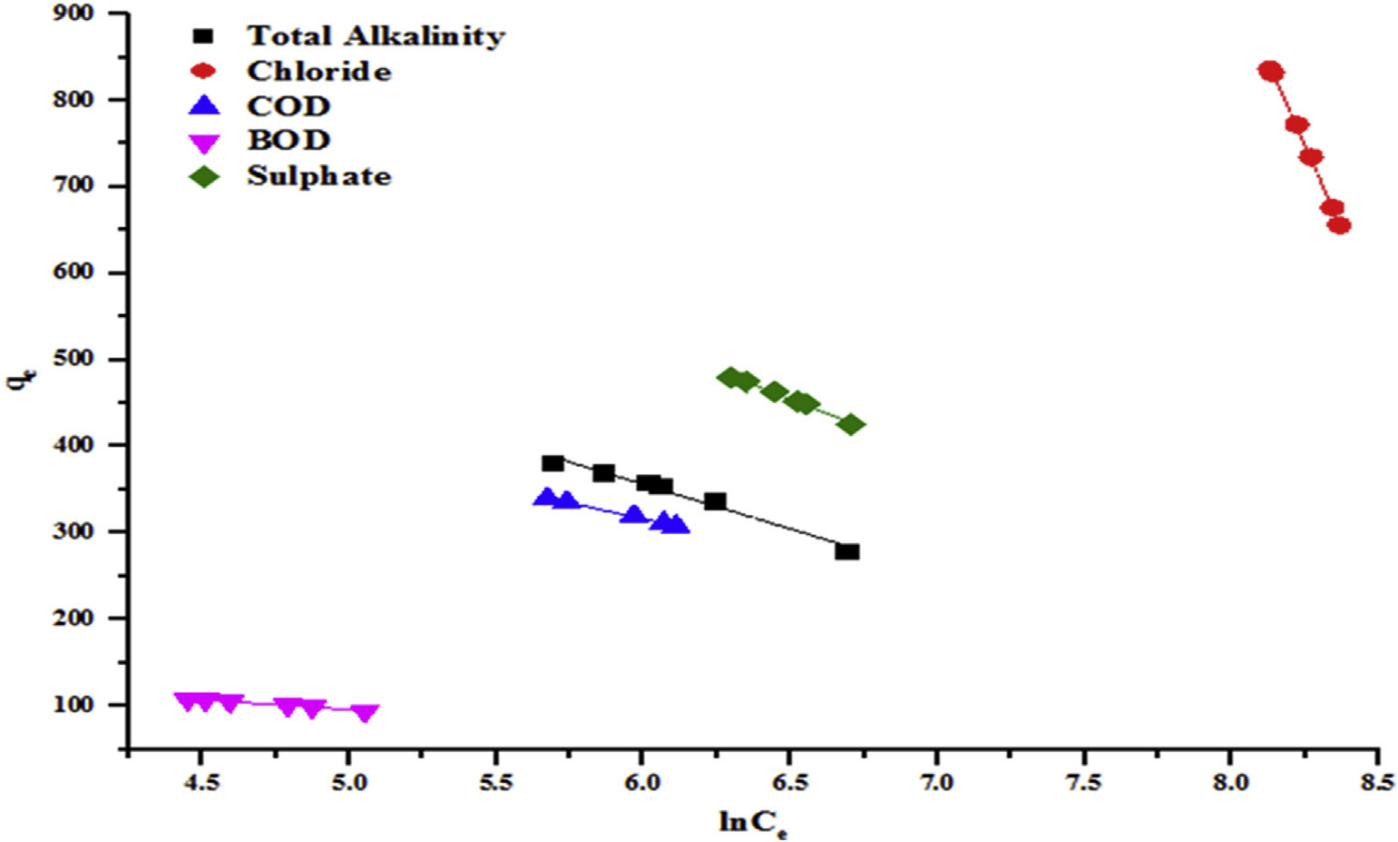
Halsey model of the removal of total alkalinity, chloride, COD, BOD and sulphate by the beneficiated kaolin at pH 5.84 and dosage, 0.2 g.

**Figure 12:**
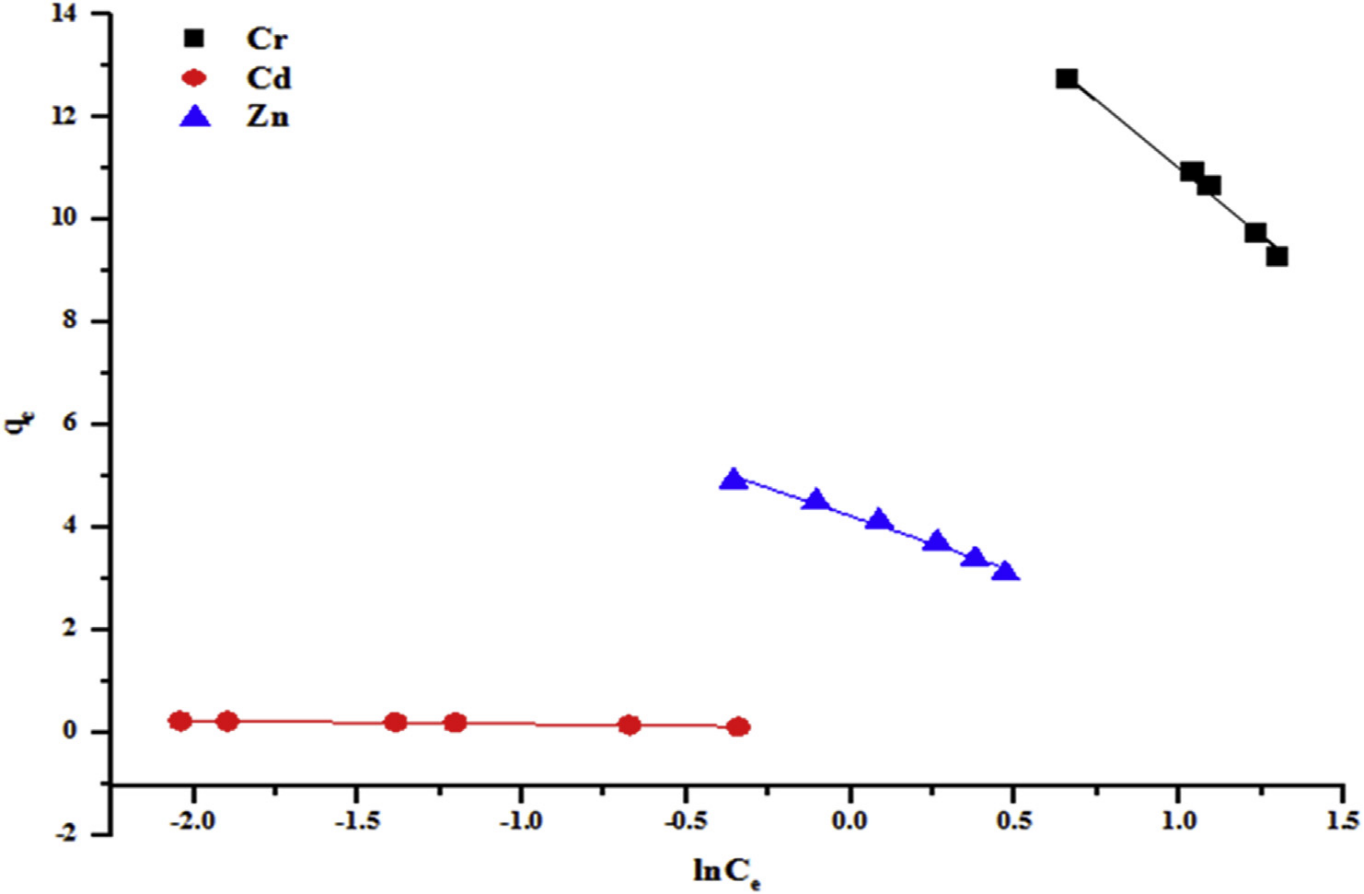
Halsey model of the removal of (a) Cr (b) Cd and (c) Zn by the beneficiated kaolin at pH 5.84 and dosage, 0.2 g.

**Figure 13:**
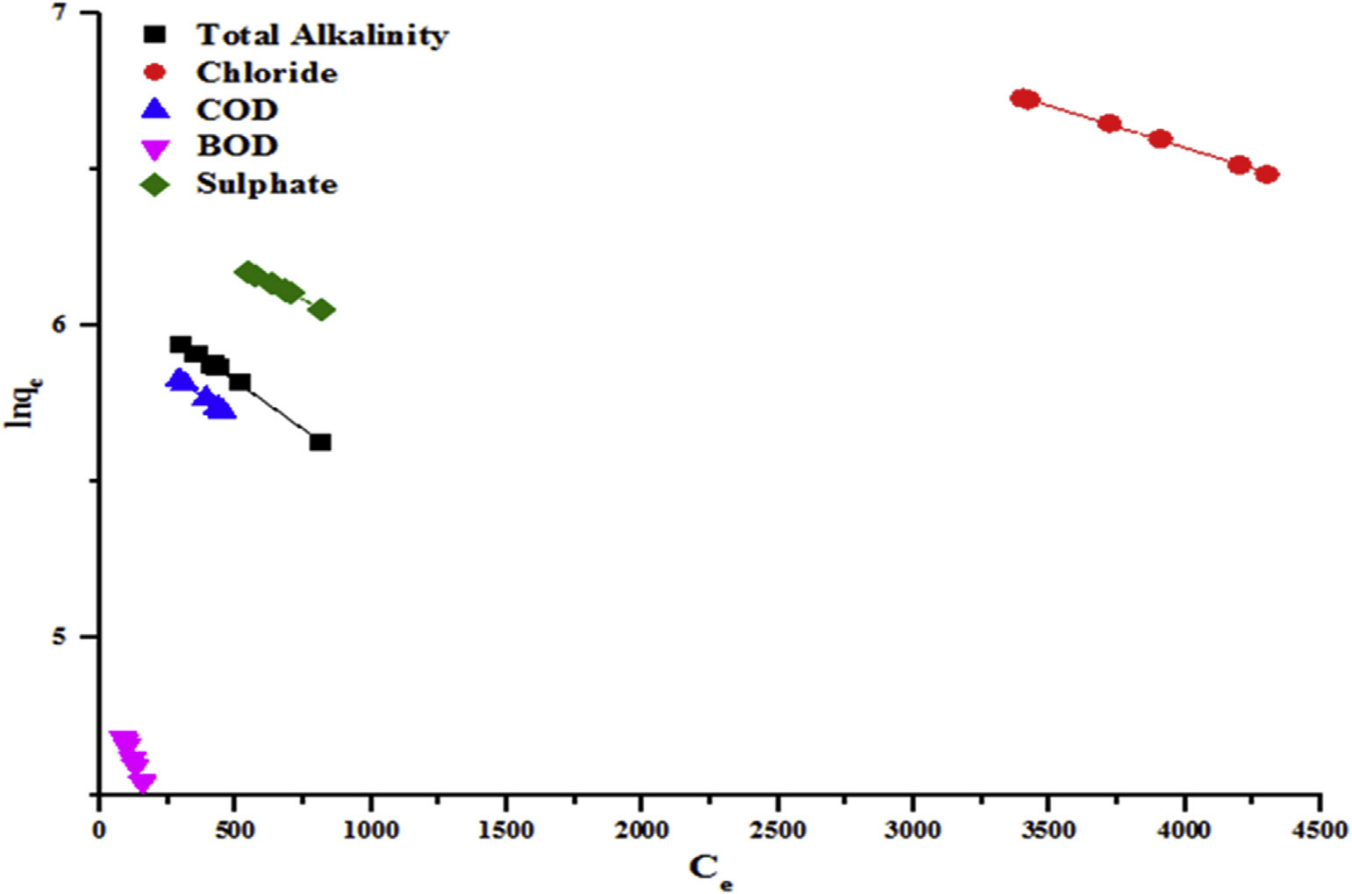
Jovanovic model for the removal of total alkalinity, chloride, COD, BOD and sulphate by the beneficiated kaolin at pH (5.84) and dosage (0.2 g).

Figures 12 and 13 present Halsey model isotherms plotted as ${\text{q}}_{\text{e}}$ against ${\text{lnC}}_{\text{e}}$ which are straight lines with slope ($\frac{1}{{\text{n}}_{\text{H}}}$) and intercept $\frac{1}{{\text{n}}_{\text{H}}}{\text{lnK}}_{\text{H}}$ for the adsorption parameters. It is evident from the plots that the linear correlation coefficient (R^2^ > 0.99) is very good i.e close to unity. The experimental data fit Halsey model which is attributed to the heterogeneous distribution of the activated sites and multilayer adsorption on kaolin. The estimated values of Halsey parameters from Figures 11 and 12 are presented in Tables 3 and 4, respectively.

#### 3.5.2 Jovanovic isotherm

Jovanovic model was employed in order to evaluate the possibility of some mechanical contacts between the sorbate and adsorbents. The linear form of the adsorption model is given as follows:

(5) $${\text{lnq}}_{\text{e}}={\text{lnq}}_{\text{e}\left(\text{max}\right)}-{\text{K}}_{\text{j}}{\text{C}}_{\text{e}}$$

Where ${\text{q}}_{\text{e}}$ the equilibrium adsorption capacity (mg/g) is, ${\text{q}}_{\text{max}}$ is the maximum adsorption capacity, (mg/g) and ${\text{K}}_{\text{j}}$ is Jovanovic constant.

**Figure 14:**
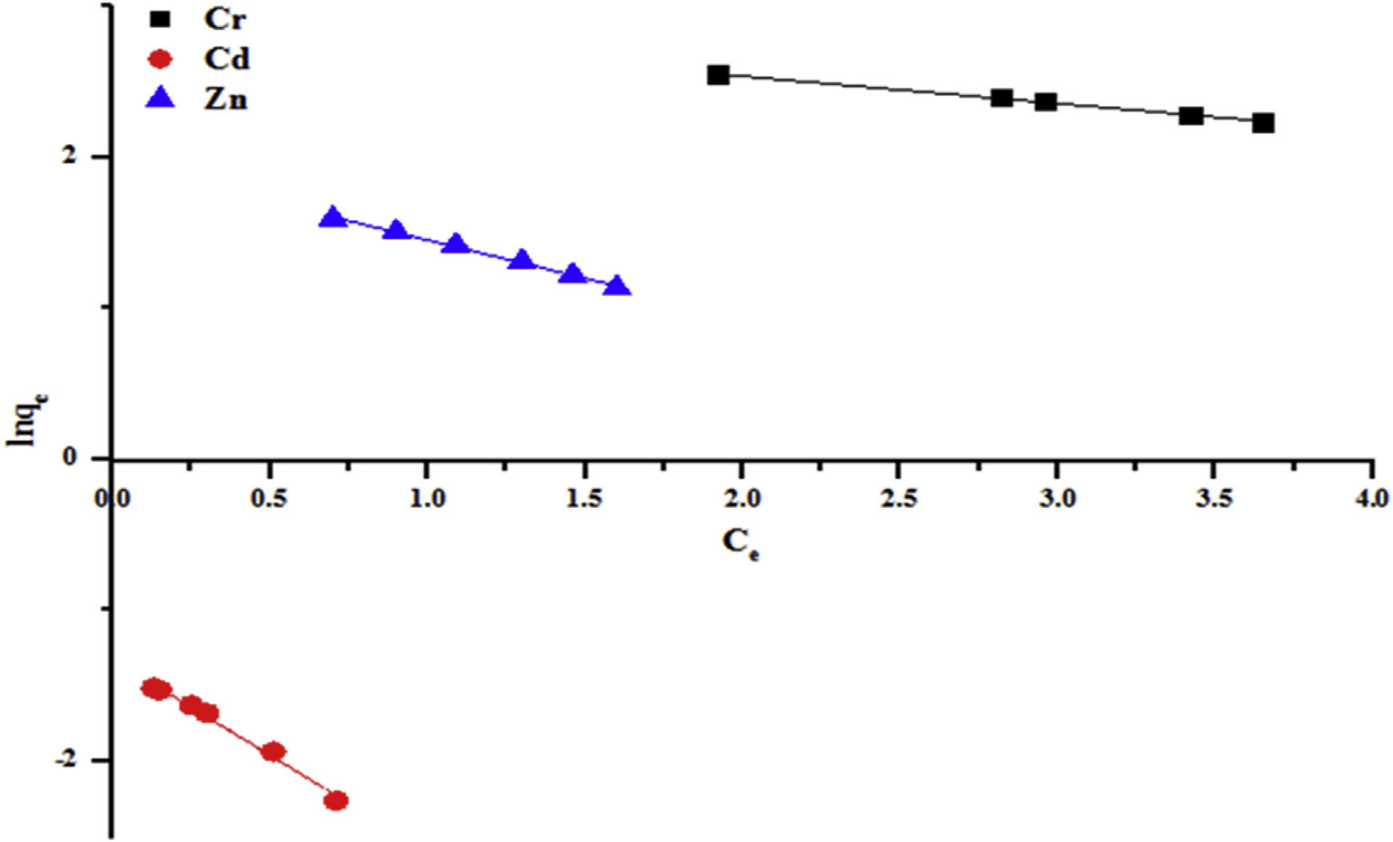
Jovanovic model for the removal of Cr, Cd and Zn by the beneficiated kaolin at pH (5.84) and dosage (0.2 g).

Figures 13 and 14 show the plots of ${\text{lnq}}_{\text{e}}$ vs $C_{e}$ for the removal of some wastewater parameters which give straight lines with slopes (${\text{K}}_{\text{j}}$) and intercepts (${\text{lnq}}_{\text{max}}$). Parameters of Jovanovic isotherms for the removal of the selected pollutants using kaolin were calculated and presented in Tables 3 and 4. The linear correlation coefficient (R^2^) ranged from 0.996 to 0.999. This confirms that the adsorption experimental data of the beneficiated kaolin follow the Jovanovic model.

The Jovanovic model showed the highest R^2^ value which indicates that the model fits the adsorption of the pollutants onto the adsorbent. This confirms that the surface of kaolin is homogeneous with monolayer adsorption on kaolin and also the possibility of some mechanical contacts between the sorbate and sorbent. This could be as a result of the presence of functional groups on the surface of kaolin as indicated by the FTIR results.

#### 3.5.3 Redlich-Peterson isotherm

The Redlich-Peterson (R–P) (1959) isotherm is a three-parameter empirical adsorption model comprising Langmuir and Freundlich isotherms. The linear expression of the R–P isotherm model is as follows:

(6) $${\text{q}}_{\text{e}}=\frac{{\text{K}}_{\text{R}}{\text{C}}_{\text{e}}}{1+{\text{ a}}_{\text{R}}{\text{C}}_{\text{e}}^{\text{g}}}$$

The linear form of the isotherm can be described as:

(7) $$\text{ln}\frac{{\text{C}}_{\text{e}}}{{\text{q}}_{\text{e}}}={\text{glnC}}_{\text{e}}-{\text{lnK}}_{\text{R}}$$

Where ${\text{K}}_{\text{R}}$ (L/g) and ${\text{a}}_{\text{R}}$ (L/mg) are the Redlich-Peterson isotherm constants and g is the exponent between 0 and 1.

**Figure 15:**
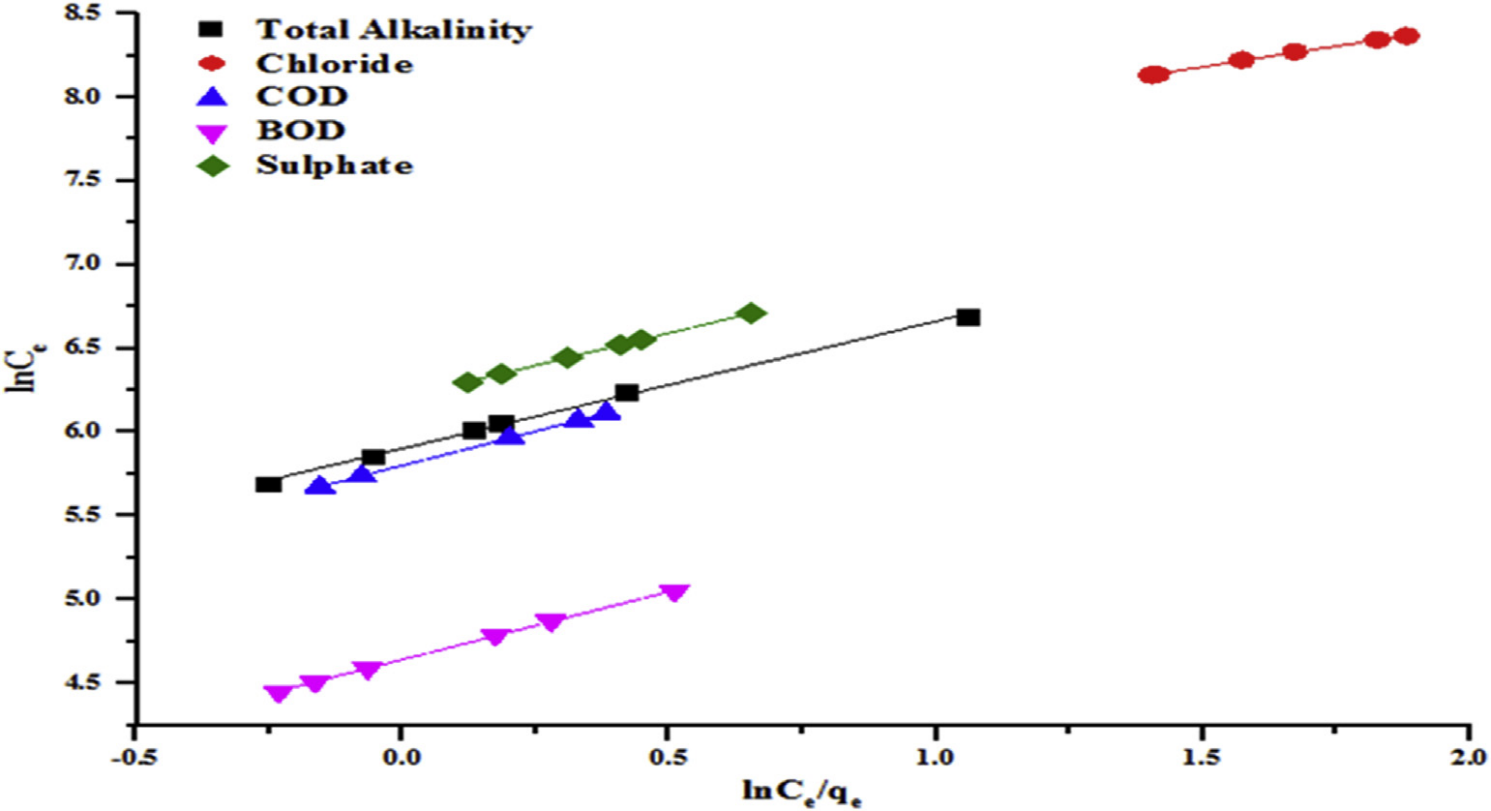
Redlich-Peterson model for the removal of (a) Total alkalinity (b) chloride (c) COD (d) BOD (e) sulphate by the beneficiated kaolin at pH (5.84) and dosage (0.2 g).

**Figure 16:**
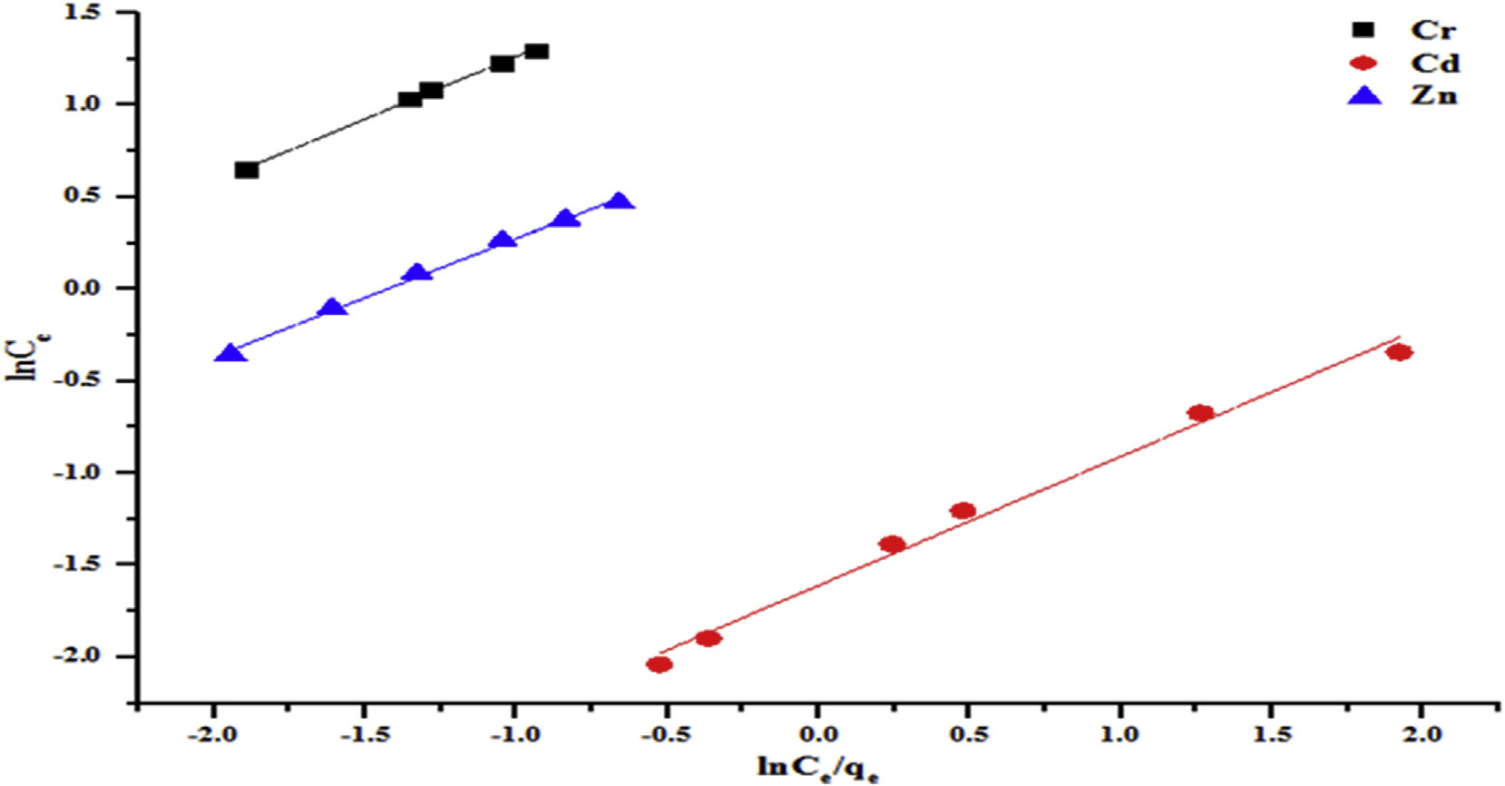
Redlich-Peterson model for the removal of (a) Cr (b) Cd and (c) Zn by the beneficiated kaolin at pH (5.84) and dosage (0.2 g).

The plots of $\text{ln}\frac{{\text{C}}_{\text{e}}}{{\text{q}}_{\text{e}}}$ against ${\text{lnC}}_{\text{e}}$ as shown in Figures 15 and 16 describe the isotherm constants g and ${\text{K}}_{\text{R}}$ for the slope and intercept, respectively. The values of ${\text{K}}_{\text{R}}$, presented in Tables 3 and 4, indicate that the adsorption capacity of the adsorbent decreased with increasing temperature. Also, the value of g lies between 0 and 1, indicating favourable adsorption.

#### 3.5.4 Flory-Huggins isotherm

The Flory-Huggins isotherm model describes the degree of surface coverage properties of the sorbate on the sorbent. The Flory-Huggins model is expressed as:

(8) $$\text{ln}\frac{\theta }{{\text{C}}_{\text{o}}}={\text{lnK}}_{\text{FH}}+\text{nln}\left(1-\theta \right)$$

(9) $$\theta =\left(1-\frac{{\text{C}}_{\text{e}}}{{\text{C}}_{\text{o}}}\right)$$

The adsorption data at different temperatures are plotted as a function of $\text{ln}\frac{\theta }{{\text{C}}_{\text{o}}}$ against ln(1−θ). Where n is the number of adsorbates occupying the adsorption sites, θ is the degree of surface coverage and ${\text{K}}_{\text{FH}}$ is the Flory-Huggins constant.

**Figure 17:**
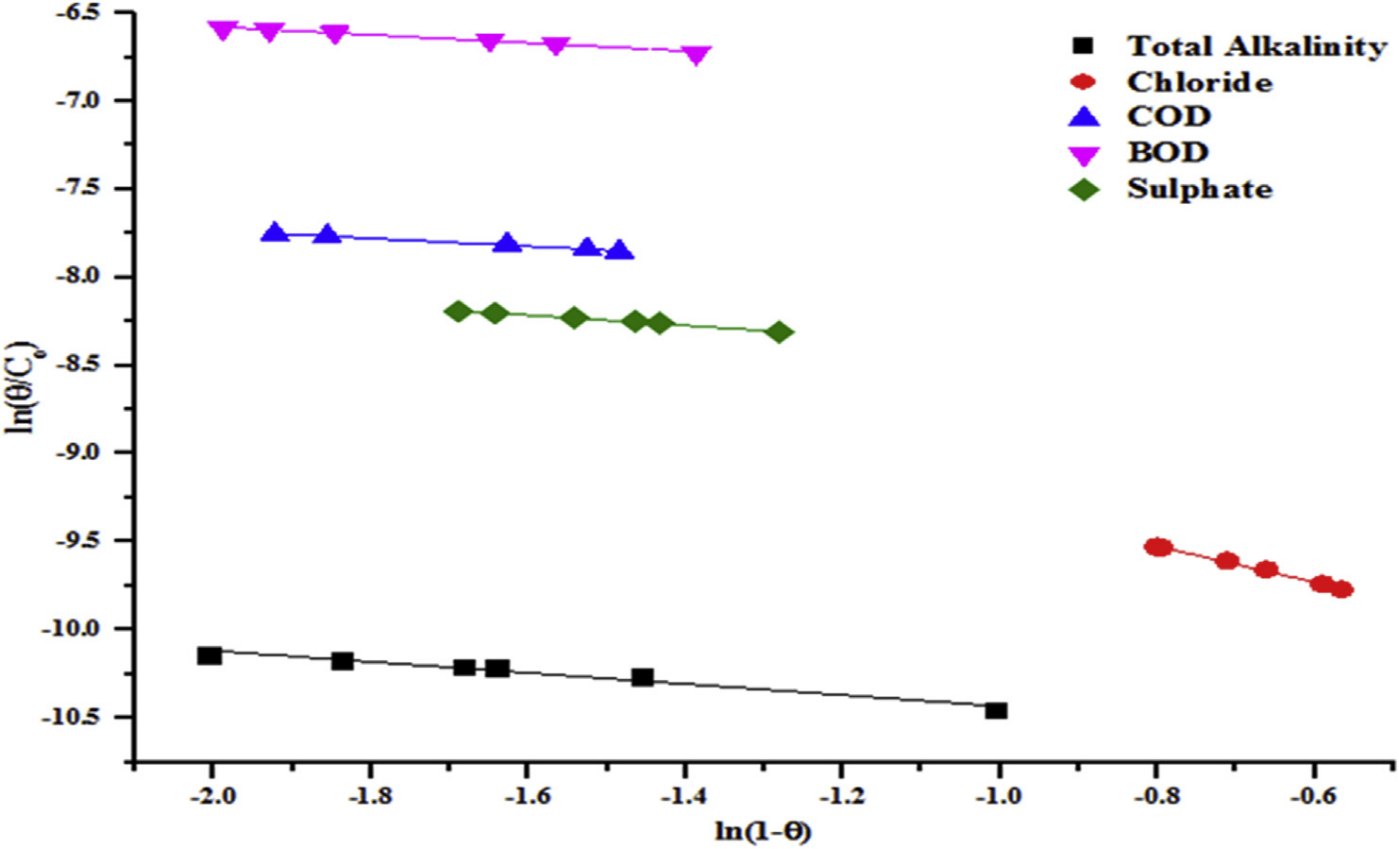
Flory-Huggins model for the removal of total alkalinity, chloride, COD, BOD and sulphate by the beneficiated kaolin at pH (5.84) and dosage (0.2 g).

**Figure 18:**
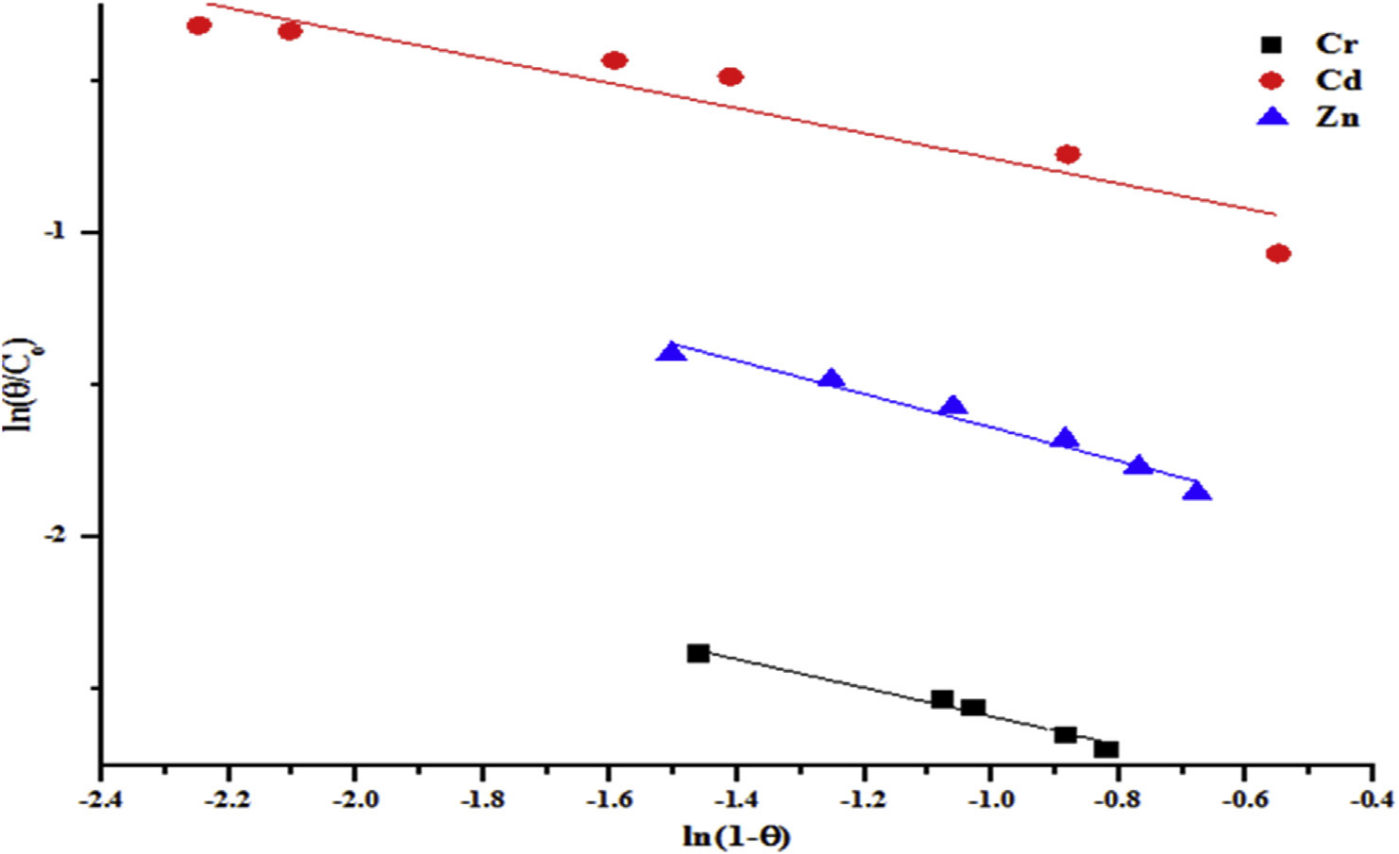
Flory-Huggins model for the removal of (a) Cr (b) Cd and (c) Zn by the beneficiated kaolin at pH (5.84) and dosage (0.2 g).

The linear form of the Flory-Huggins isotherm model presents the Flory-Huggins equilibrium constant (K_FH_) as presented in Tables 3 and 4. The isotherm model showed the feasibility and spontaneity of the adsorption process. Using Eq. (10) and the plots of Figures 17 and 18 to compute the spontaneity Gibb's free energy, it showed that the ΔG is negative. This indicates the influence of temperature during the adsorption system.

(10) $$\Delta \text{G}=\text{RTln}\left({\text{K}}_{\text{FH}}\right)$$

Where ΔG the free energy change (kJ/mol), R is the universal gas constant, (8.314 J/molK), T is the absolute temperature, (K) and Flory-Huggins equilibrium constant, K_FH_.

### 3.6 Kinetic models

The kinetic analysis predicts the mechanism involved in adsorption and points out the rate-limiting step of the process. The kinetic data were analyzed using the Bangham, fractional power and Avrami equation models. The Boyd kinetic equation and Weber-Morris intraparticle diffusion models were used to check the mechanism of adsorption.

Bangham's model equation is expressed as:

(11) $$\log\left[\log\left(\frac{{\text{C}}_{\text{o}}}{{\text{C}}_{\text{o}}-{\text{q}}_{\text{t}}\text{M}}\right)\right]=\log\left(\frac{{\text{K}}_{\text{o}}\text{M}}{2.303\text{V}}\right)+\alpha \text{logt}$$

Where ${\text{C}}_{\text{o}}$ is the initial concentration of the pollutant in wastewater solution (mg/dm^3^), V is the volume of wastewater solution (dm^3^), M is the weight of adsorbent (g/dm^3^) qt is the amount of pollutant adsorbed at the time, t (mg/g) and ${\text{K}}_{\text{o}}$ and α (less than 1) are constants which are calculated from the intercept and slope of the linear plot of $\log\left[\log\left(\frac{{\text{C}}_{\text{o}}}{{\text{C}}_{\text{o}}-{\text{q}}_{\text{t}}\text{M}}\right)\right]$ against logt.

The Bangham kinetic model was used to observe the pore diffusion in the adsorption system and the α and Bangham constant are presented in Tables 5 and 6. The extrapolation of the double logarithm order kinetic model show that multiple adsorption stages occurred and the correlation coefficient obtained for the model ranged between 0.6421 and 0.99248, showing that pore diffusion was involved in the pollutant removal using the beneficiated kaolin and the nanocomposites. On the other hand, the linearity of the Bangham plot indicates that the sorbate pore diffusion is not the solitary rate-controlling step.

**Table 5:** Kinetic model constants for BOD, total alkalinity, COD, chloride and sulphate adsorption onto kaolin a dosage (0.2 g), pH (5.84) and temperature (298 K).

Parameter		Parameter				
		Total Alkalinity	Chloride	COD	BOD	Sulphate
Bangham	α	0.1157	0.0933	0.0529	0.0543	0.0568
	${\text{K}}_{\text{i}}$	4.916	4.076	4.935	5.527	4.843
	${\text{R}}^{2}$	0.87305	0.95698	0.90401	0.88789	0.94678
Avrami	${\text{n}}_{\text{AV}}$	0.574	0.496	0.229	0.272	0.247
	${\text{K}}_{\text{AV}}$	-0.145	-2.256	-0.119	-1.597	-0.454
	${\text{R}}^{2}$	0.91642	0.98299	0.90502	0.87159	0.94758

**Table 6:** Kinetic model constants for Cr, Cd and Zn adsorption onto kaolin a dosage (0.2 g), pH (5.84) and temperature (298 K).

Parameter		Parameter		
		Chromium	Cadmium	Zinc
Bangham	α	0.025	0.234	0.163
	${\text{K}}_{\text{i}}$	3.877	2.563	3.105
	${\text{R}}^{2}$	0.71904	0.84886	0.92607
Avrami	${\text{n}}_{\text{AV}}$	0.520	0.863	0.278
	${\text{K}}_{\text{AV}}$	-2.165	-1.981	-2.098
	${\text{R}}^{2}$	0.84700	0.77477	0.88305

The Avrami equation is used to evaluate the kinetic parameters as functions of contact times (0–30 min). The linear form of the Avrami kinetic model is expressed as:

(12) $$\ln\left[\ln\left(\frac{{\text{q}}_{\text{e}}}{{\text{q}}_{\text{e}}-{\text{q}}_{\text{t}}}\right)\right]={\text{n}}_{\text{AV}}{\text{lnK}}_{\text{AV}}+{\text{n}}_{\text{AV}}\text{lnt}$$

Where ${\text{K}}_{\text{AV}}$ is the Avrami kinetic constant and ${\text{n}}_{\text{AV}}$ is the Avrami model constant related to the adsorption mechanism. The values of ${\text{K}}_{\text{AV}}$ and ${\text{n}}_{\text{AV}}$ are obtained for the intercept and slope, respectively, from the plot of $\ln\left[\ln\left(\frac{{\text{q}}_{\text{e}}}{{\text{q}}_{\text{e}}-{\text{q}}_{\text{t}}}\right)\right]$ versus lnt.

The Avrami kinetic parameters; kinetic rate constant (K_AV_), Avrami exponent (A_AV_) and correlation coefficient (R^2^) are presented in Tables 5 and 6. The A_AV_ values are positive in all the pollutants uptake onto the adsorbent sample. This shows that there is a possible change in the adsorption process. There is also an indication that during the contact of the sorbate with the adsorbents the rate of adsorption could follow multiple kinetic orders.

The kinetic data suitably fitted well to the Avrami kinetic model that presented the highest R^2^ values for all the studied pollutants using the beneficiated kaolin when compared to Bangham.

### 3.7 Adsorption thermodynamic

The adsorption system takes into account the mechanism involved based on thermodynamics. The Gibbs free energy, enthalpy and entropy at different temperatures were calculated from the following equations:

(13) $$\Delta \text{G}=-\text{RTln}\left({\text{K}}_{\text{D}}\right)$$

(14) $${\text{lnK}}_{\text{D}}=\frac{\Delta \text{S}}{\text{R}}-\frac{\Delta \text{H}}{\text{RT}}$$

Where ΔG is the free energy change (kJ/mol), ΔS is the change in entropy (J/molK), ΔH is the change in enthalpy (J/mol), R is the universal gas constant (8.314 J/molK), T is the absolute temperature (K) and ${\text{K}}_{\text{D}}$ is the thermodynamic equilibrium constant. The value of ${\text{K}}_{\text{D}}$ is calculated from the relation as shown in Eq. (13).

(15) $${\text{K}}_{\text{D}}=\frac{{\text{C}}_{\text{o}}-{\text{C}}_{\text{e}}}{{\text{C}}_{\text{e}}}\times \frac{\text{V}}{\text{M}}$$

The ΔG is calculated according to the following equation:

(16) ΔG=ΔH−TΔS

The values of thermodynamic parameters are shown in Tables 7 and 8. The positive values of ΔH from the adsorption system indicates that the adsorption is an endothermic process, which explains the fact that the adsorption process increased with increase in temperature. The positive values of ΔS for all the pollutants show an increase in randomness at the solid-liquid interface during the adsorption process. The results (Tables 7 and 8) show that the ΔG is high at low temperature, indicating less favorability of the adsorption process. The rate of adsorption is lower with increasing temperature. This indicates that the adsorption process becomes more negative at high temperature. The thermodynamic study reveals that the sorption process of pollutants onto kaolin is spontaneous for Zn and Cr while it is non-spontaneous in nature for Cd. Therefore, increasing the temperature favour more adsorption process for the parameters that were studied.

**Table 7:** Thermodynamic parameters for the adsorption of BOD, total alkalinity, COD, chloride and sulphate from tannery wastewater solution unto kaolin.

Metal	T(K)	ΔG(kJ/mol)	ΔH(kJ/mol)	ΔS(J/molK)	$R^{2}$
TA	303	2.544	20.647	61.066	0.82583
	313	1.533			
	323	0.929			
	333	0.312			
	343	-0.299			
	353	-0.909			
BOD	303	1.205	13.47	40.480	0.97300
	313	0.800			
	323	0.395			
	333	0.00984			
	343	-0.415			
	353	-0.819			
COD	303	1.0960	9.827	28.816	0.88560
	313	0.808			
	323	0.519			
	333	0.231			
	343	0.0569			
	353	-0.345			
Chloride	303	4.802	15.456	9.485	0.96286
	313	4.647			
	323	4.493			
	333	4.338			
	343	4.184			
	353	4.029			
Sulphate	303	1.559	9.038	24.684	0.96578
	313	1.312			
	323	1.065			
	333	0.818			
	343	0.571			
	353	0.325

**Table 8:** Thermodynamic parameters for the adsorption of zinc, cadmium and chromium ions from tannery wastewater solution unto kaolin.

Metal	T(K)	ΔG(kJ/mol)	ΔH(kJ/mol)	ΔS(J/molK)	$R^{2}$
Zinc	303	-1.372	21.096	74.153	0.91998
	313	-2.114			
	323	-2.855			
	333	-3.597			
	343	-4.338			
	353	-5.080			
Cadmium	303	3.624	35.713	105.904	0.93850
	313	2.588			
	323	1.506			
	333	0.447			
	343	-0.612			
	353	-0.670			
Chromium	303	-2.151	18.725	68.898	0.90041
	313	-2.840			
	323	-3.529			
	333	-4.218			
	343	-4.902			
	353	-5.596			

### 3.8 Comparative study

Several studies on the use of clay for the removal of pollutants have been done in various parts of Nigeria. The availability of this substance as an adsorbent in Nigeria along with its locations and experimental techniques employed are listed in Table 9. In the present study, kaolin was used for the removal of pollutants in tannery wastewater and compared with using clay from various parts of Nigeria as reported in the literature. Table 9 shows that the insight gained from adsorption studies using this adsorbent in different forms especially on a nanoscale, similar to what was done in this study, will serve as an additional source of information in the literature. Thus, the use of natural adsorbents, such as kaolin, for the removal of pollutants in industrial wastewater, will provide an environmental benefit for industries. This is because even from this study, it has been established that the use of natural kaolin like that from Gbako Local Government Area, Niger State, Nigeria has proven to be a good alternative for industrial wastewater treatment. It has been found to be an effective, economical and environmentally friendly approach for the removal of pollutants from industrial wastewaters.

**Table 9:** List of Nigerian natural clays used as adsorbents for water treatment.

Adsorbent	Location/State	Experiment	Solution	Reference
Kaolin	Aloij, Kogi	Batch adsorption	Simulated wastewater (Ni and Mn)	Dawodu and Akpomie (2014)
Kaolin	Mba-ano, Imo	Batch adsorption	River water	Agbo et al. (2015)
Bentonite and kaolin	Afashio, Edo	Batch adsorption	Palm oil	Usman et al. (2013)
Kaolin	Ehime-Mbano, Imo	Coagulation and adsorption	Industrial paint	Nwuzor et al. (2018)
Kaolin	PRODA, Enugu	Batch adsorption	Simulated wastewater (Zn)	Ekekwe et al. (2018)
Kaolin	Okefomo, Agbarohidoma, Kwara	Coagulation		Kuranga et al. (2018)
Kaolin	Isuija, Enugu	Batch adsorption	Crude oil	Akpomie et al. (2019)
Montmorillonite	Oji, Enugu	Batch adsorption	Automobile effluent (Zn, Cu, Mn, Cd and Pb)	Akpomie et al. (2015)
Kaolin	Aloji. Kogi	Batch adsorption	Simulated wastewater (Pb and Cd)	Ogbu et al. (2019)
Kaolin	Aloji, Kogi	Batch adsorption	Simulated wastewater (Pb and Cd)	Chukwuemeka-Okorie et al. (2018)
Kaolin	Mowe, Ogun	Batch adsorption	Simulated wastewater (Pb, Cd and Ni)	Unuabonah et al. (2013)
Kaolin	Ire-Ekiti, Ekiti	Batch adsorption	Simulated wastewater (Pb, Cr, Ni and Cu)	Kayode et al. (2019)
Kaolin	Ubulu-Ukwu, Delta	Batch adsorption	Simulated wastewater (Pb and Cd)	Unuabonah et al. (2008)
Clay	Ekiti	Batch adsorption	Simulated wastewater (Pb, Cu, Cd and Zn)	Olaofe et al. (2015)
Kaolin	PRODA, Enugu	Batch adsorption	Simulated wastewater (Pb)	Orumwense (1996)
Montmorillonite	Ugwuoba, Enugu	Batch adsorption	Automobile (Zn, Cu, Mn, Cd, Pb and Ni)	Akpomie and Dawodu (2016)
Montmorillonite	Edo	Batch adsorption	Pharmaceutical	Egbon et al. (2013)
Kaolin	Gbako, Niger	Batch adsorption	Tannery wastewater	Present study

### 3.9 Economic evaluation and future directions

This study demonstrates the promising nature of the use of beneficiated kaolin for the removal of pollutants. Modifications carried out on the kaolin may increase the adsorption capacity of this readily available inexpensive natural material that has several industrial applications. Thus, this present study would serve as a good reference point for harnessing kaolin as a useful industrial material in the industrial wastewater purification processes. This is because kaolin in modified forms is very effective for adsorbing pollutant from wastewater. It is abundant in nature, has low-cost and has a high specific surface area. This naturally available substance can also be used in conjunction with other materials in order to enhance their adsorption capacities. Since it was reported by Zen et al. (2018) and Marino et al. (2017) that modified kaolin and membrane separation combined with adsorption process using kaolin are promising adsorbents for wastewater treatment, respectively; thus, this may lead to their widespread use in innovative technologies.

## 4 Conclusion

In this current research, kaolin obtained from Gbako Local Government in Niger State, Nigeria was used as an adsorbent for the removal of total alkalinity, chloride, COD, BOD, sulphate, Cr, Cd and Zn. The kaolin structure was characterized using XRD, FTIR, HRSEM, HRTEM and BET. Batch adsorption techniques used in this study show that beneficiated kaolin significantly affected the contact time, adsorbent dosage and temperature of wastewater. It was found that the adsorption capacity of kaolin increases with adsorbent dosage and temperature; therefore, the removal of the parameters by kaolin was temperature-dependent. The isotherm and kinetic models for the adsorption of the pollutants from wastewater conform to the Jovanovic and Redlich-Peterson isotherm models based on the correlation coefficients (R^2^) best fit the experimental data obtained. Based on the comparison of the Bangham and Avrami kinetic models, the Avrami model provided a better correlation coefficient for the adsorption of the pollutants. More so, charged surface, water absorption process and surface morphology of kaolin could contribute to the uptake of the pollutants from wastewater. Thus, these results provide additional information on the uses of kaolin found in Nigeria which is quite cheap and non-hazardous to the environment in wastewater treatment.

## Declarations

### Author contribution statement

Mustapha S: Conceived and designed the experiments; Performed the experiments; Analyzed and interpreted the data; Wrote the paper.

Ndamitso M.M, Abdulkareem A.S, Tijani J.O: Conceived and designed the experiments; Contributed reagents, materials, analysis tools or data.

Mohammed A.K, Shuaib D.T: Analyzed and interpreted the data.

### Funding statement

This work was supported by the Tertiary Education Trust Fund (TETFund) of Nigeria under a grant number TETFUND/FUTMINNA/2017/01.

### Competing interest statement

The authors declare no conflict of interest.

### Additional information

No additional information is available for this paper.
